# Macrophage acetyl-CoA carboxylase regulates acute inflammation through control of glucose and lipid metabolism

**DOI:** 10.1126/sciadv.abq1984

**Published:** 2022-11-23

**Authors:** Scott Yeudall, Clint M. Upchurch, Philip V. Seegren, Caitlin M. Pavelec, Jan Greulich, Michael C. Lemke, Thurl E. Harris, Bimal N. Desai, Kyle L. Hoehn, Norbert Leitinger

**Affiliations:** ^1^ Department of Pharmacology, University of Virginia School of Medicine, Charlottesville, VA 22908, USA.; ^2^ Robert M. Berne Cardiovascular Research Center, University of Virginia School of Medicine, Charlottesville, VA 22908, USA.; ^3^ Environmentally-Induced Cardiovascular Degeneration, Clinical Chemistry and Laboratory Diagnostics, Medical Faculty, University Hospital and Heinrich-Heine University Düsseldorf, 40225 Düsseldorf, Germany.; ^4^ School of Biotechnology and Biomolecular Sciences, University of New South Wales, Sydney, New South Wales 2052, Australia.

## Abstract

Acetyl-CoA carboxylase (ACC) regulates lipid synthesis; however, its role in inflammatory regulation in macrophages remains unclear. We generated mice that are deficient in both ACC isoforms in myeloid cells. ACC deficiency altered the lipidomic, transcriptomic, and bioenergetic profile of bone marrow–derived macrophages, resulting in a blunted response to proinflammatory stimulation. In response to lipopolysaccharide (LPS), ACC is required for the early metabolic switch to glycolysis and remodeling of the macrophage lipidome. ACC deficiency also resulted in impaired macrophage innate immune functions, including bacterial clearance. Myeloid-specific deletion or pharmacological inhibition of ACC in mice attenuated LPS-induced expression of proinflammatory cytokines interleukin-6 (IL-6) and IL-1β, while pharmacological inhibition of ACC increased susceptibility to bacterial peritonitis in wild-type mice. Together, we identify a critical role for ACC in metabolic regulation of the innate immune response in macrophages, and thus a clinically relevant, unexpected consequence of pharmacological ACC inhibition.

## Teaser

Loss of ACC blunts inflammatory macrophage polarization by impairing stimulus-induced change in glucose and lipid metabolism.

## INTRODUCTION

Stimulus-dependent metabolic reprogramming is a hallmark of phenotypic polarization of immune cell types including macrophages (*1*). In response to Toll-like receptor (TLR)–activating pathogen-derived danger signals such as lipopolysaccharide (LPS), M1 macrophages rapidly switch to aerobic glycolysis for adenosine triphosphate (ATP) production and increase glucose uptake, and blockade of either of these processes is sufficient to inhibit the inflammatory response (*2*, *3*). Conversely, alternative activation (M2) of macrophages with stimuli such as interleukin-4 (IL-4) demonstrates a reliance on oxidation of fatty acids to fuel increased oxidative phosphorylation (*4*, *5*). Moreover, exposure of macrophages to redox-modulatory stimuli such as lipid oxidation products (Mox) or cell-free heme (Mhem) results in increased flux through the pentose phosphate pathway and production of NADPH [reduced form of nicotinamide adenine dinucleotide phosphate (NADP^+^)] and the reducing agent glutathione at the expense of glycolysis (*6*, *7*).

A defining hallmark of the inflammatory response in macrophages is the accumulation of intracellular lipids, which is dependent on activation of TLR signaling (*8*). Genetic ablation of fatty acid synthase (FAS) in macrophages blunts proinflammatory polarization, adipose tissue inflammation, and insulin resistance in response to high-fat feeding through perturbations in membrane composition and LPS-induced signaling (*9*), while pharmacologic inhibition of FAS dampens dendritic cell activation in response to LPS by blocking endoplasmic reticulum (ER) and Golgi expansion (*10*). In addition, loss of diacylglycerol acyltransferase (DGAT)–dependent triacylglycerol (TAG) production in macrophages attenuates LPS-induced inflammation, in part through alterations in prostaglandin production (*11*). Furthermore, activation of the sterol- and lipid-sensing transcription factors liver X receptor (LXR) and sterol regulatory element–binding protein 1 (SREBP1) is critical for the resolution of TLR-dependent inflammation (*12*, *13*).

Acetyl–coenzyme A (CoA) carboxylase (ACC) enzymes, which catalyze the conversion of acetyl-CoA to malonyl-CoA committing carbon to de novo fatty acid synthesis, are central regulators of lipid metabolism in the cell. Mammals encode two isoforms of ACC: ACC1 is a cytosolic enzyme that acts to generate the malonyl-CoA primarily as a substrate for the lipogenesis pathway, while the localized production of malonyl-CoA by ACC2 acts to inhibit acylcarnitine transport into the mitochondria via carnitine palmitoyltransferase 1 (CPT1) (*14*), thereby regulating β-oxidation of fatty acids (*15*). Although the two isoforms are thought to serve these distinct roles, they catalyze the same biochemical transformation and are regulated at the enzymatic level by similar mechanisms such as phosphorylation (*16*–*18*). Whole-animal deletion of ACC1 is lethal during development (*19*), whereas global ACC2 knockout mice are viable (*20*). Some studies of global ACC2 knockout mice demonstrated increased mitochondrial oxidation, lower fat stores, and increased insulin sensitivity (*21*, *22*), while in other studies ACC2 mutant mice had increased levels of fatty acid oxidation without changes in adiposity or energy balance (*23*). Previous studies have demonstrated that liver-specific deletion of only ACC1 can result in compensation by the ACC2 isoform (*24*). However, genetic deletion of both ACC1 and ACC2 resulted in increased cellular glucose metabolism in hepatocytes in vitro and increased hepatic lipid accumulation in vivo. Unexpectedly, ablation of ACC expression also caused a shift in the global profile of protein acetylation, without significantly altering overall levels of acetyl-CoA (*25*). The role for ACC enzymes in immune cells, however, remains less clear: Individual deletion of ACC1 or ACC2 in the myeloid lineage had minimal impact on the inflammatory response to mycobacterial infection (*26*). However, the consequences of ACC1/ACC2 double deletion, which eliminates potential for compensatory enzymatic activity, on myeloid cell function have not been assessed.

Here, we examined the impact of ACC deficiency on macrophage phenotypic and metabolic polarization during the inflammatory response. Using mice with a myeloid-specific deletion of both ACC isoforms, we demonstrate that ACC knockdown attenuates the LPS-induced macrophage activation through perturbation of cellular glucose and lipid metabolism to blunt inflammatory cytokine induction and impair macrophage effector functions. These findings identify a critical role for ACC enzymes at the nexus of metabolism, lipid synthesis, and cellular function in macrophages and discover that attenuation of the macrophage inflammatory response is an unexpected consequence of ACC inhibition.

## RESULTS

### ACC knockdown shifts macrophages to a hyperglycolytic bioenergetic state

**Fig. 1.**
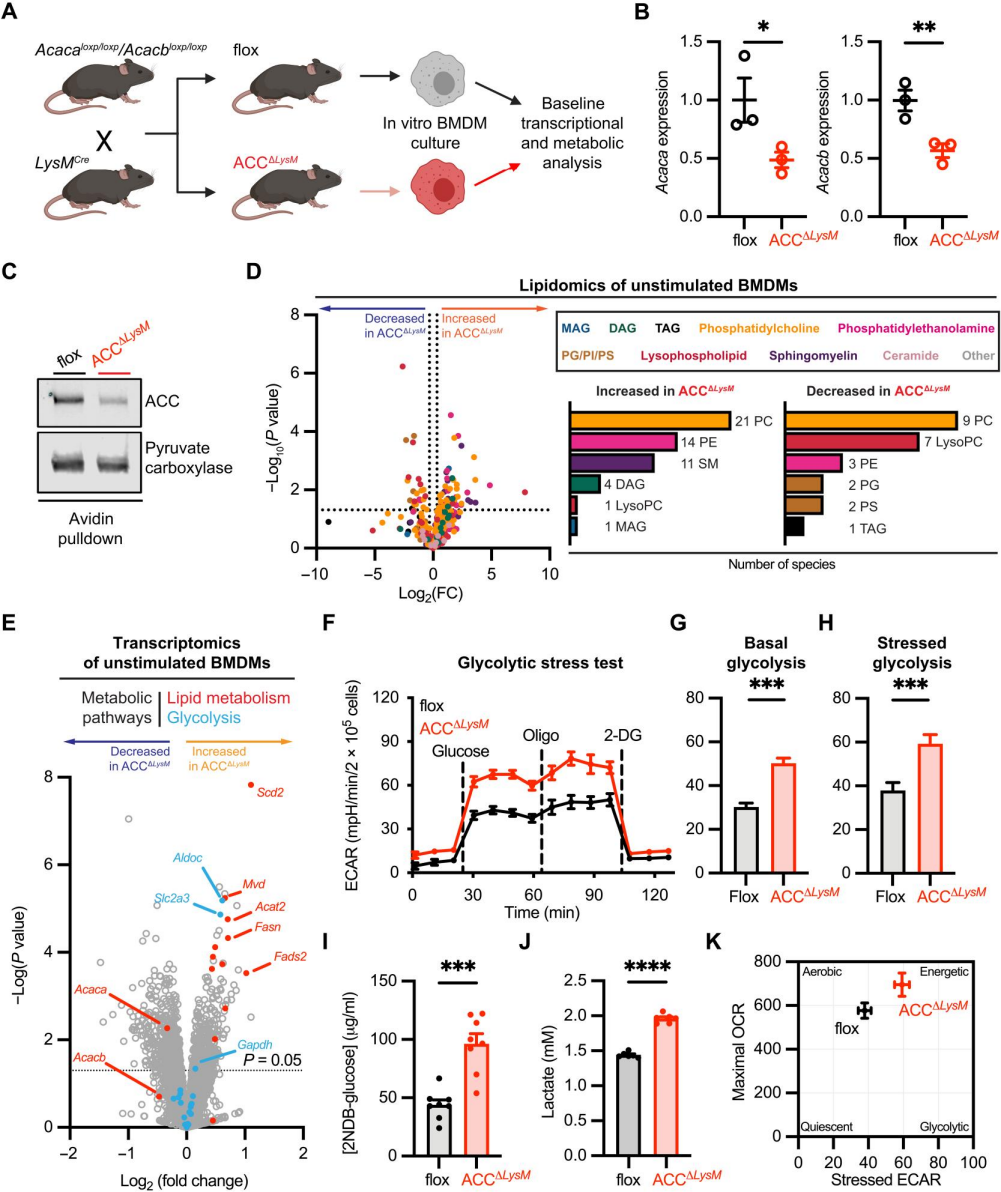
Deletion of ACC shifts macrophage metabolism to hyperenergetic, glycolytic state. (**A**) Mice carrying loxP-flanked alleles of *Acaca* and *Acacb* were crossed with *LysM*^Cre^ mice to generate mice with a myeloid-specific deletion of ACC (ACC^Δ*LysM*^). (**B**) Relative mRNA levels of *Acaca* and *Acacb* in BMDMs from flox or ACC^Δ*LysM*^ mice (*n* = 3). (**C**) Representative immunoblot of ACC after avidin pulldown of biotin-containing proteins from flox or ACC^Δ*LysM*^ BMDMs. Pyruvate carboxylase was used as a loading control. (**D**) Relative levels of lipid species in unstimulated BMDMs from flox and ACC^Δ*LysM*^ mice. Left: Number of lipids increased [fold change (FC) > 1.25] in unstimulated BMDMs from ACC^Δ*LysM*^ mice. Right: Number of lipids decreased (fold change < 1.25) in BMDMs from ACC^Δ*LysM*^ mice (*n* = 6). TAG, triacylglycerol; PE, phosphatidylethanolamine; PC, phosphatidylcholine; MAG, monoacylglycerol; SM, sphingomyelin; PS, phosphatidylserine; PI, phosphatidylinositol; LysoPC, lysophosphatidylcholine; DAG, diacylglycerol. (**E**) Volcano plot of RNA-seq from unstimulated flox or ACC^Δ*LysM*^ BMDMs (*n* = 4). (**F**) Glycolytic stress test of flox or ACC^Δ*LysM*^ BMDMs (*n* = 5). 2-DG, 2-deoxyglucose. (**G**) Basal glycolytic rate of flox or ACC^Δ*LysM*^ BMDMs (*n* = 5). (**H**) Stressed glycolytic rate of flox or ACC^Δ*LysM*^ BMDMs (*n* = 5). (**I**) Six-hour 2NDB-glucose uptake in flox or ACC^Δ*LysM*^ BMDMs (*n* = 8). (**J**) Lactate levels in supernatant of unstimulated flox or ACC^Δ*LysM*^ BMDMs after 6 hours (*n* = 6). (**K**) Bioenergetics phenogram reveals a shift to a more energetic phenotype in ACC^Δ*LysM*^ BMDMs. Data are represented as means ± SEM. Significance determined by one-tailed Welch’s *t* test (B and G to J) or two-tailed Student’s *t* test (D). *P < 0.05, **P < 0.01, and ***P < 0.001. (A) Created using BioRender.com. See also fig. S1, related to Fig. 1.

To assess the role of ACC in the macrophage inflammatory response, we crossed mice expressing Cre recombinase under the control of the myeloid-specific *LysM* promoter (*27*) with mice expressing loxP-flanked alleles of both *Acaca* (encoding ACC1) and *Acacb* (encoding ACC2) (*25*) to generate mice with a myeloid-specific knockdown of ACC1 and ACC2 (hereafter ACC^Δ*LysM*^) (Fig. 1A and fig. S1A). We confirmed significantly decreased mRNA expression of *Acaca* and *Acacb* (Fig. 1B) and decreased ACC protein levels (Fig. 1C) in bone marrow–derived macrophages (BMDMs) isolated from ACC^Δ*LysM*^ mice. The degree of knockdown was in accordance with previous studies using LysM Cre to deplete the related metabolic enzyme ATP citrate lyase (ACLY) (*28*). ACC^Δ*LysM*^ mice grew and developed normally, and we observed no difference in total body mass or fat mass in male or female mice as compared to littermate flox controls (fig. S1, B and C).

To examine the impact of decreased ACC levels on macrophages, we first assessed baseline transcriptional and metabolic profiles in vitro. Comparison of unstimulated ACC^Δ*LysM*^ and control BMDMs using mass spectrometry–based lipidomics (Fig. 1D) revealed expected changes in the lipidome of ACC-deficient macrophages, with 52 species increased in abundance and 24 species decreased in abundance in ACC^Δ*LysM*^ (Fig. 1D, insets). We then examined whether loss of ACC has any impact on the transcriptional profile of macrophages at baseline. RNA sequencing (RNA-seq) analysis of BMDMs from control or ACC^Δ*LysM*^ mice revealed changes in metabolic pathways and lipid metabolism (Fig. 1E). Gene Ontology (GO) analysis identified enrichment of processes including “cholesterol biosynthetic process,” “sterol biosynthetic process,” and “phospholipid biosynthetic process” in ACC-deficient BMDMs (fig. S1D), which also demonstrated slightly higher levels of FASN protein compared with flox controls (fig. S1E).

Further analysis of gene expression in other metabolic pathways demonstrated alterations in glycolysis and glucose metabolism (Fig. 1E and fig. S1F), which we confirmed by qualitative polymerase chain reaction (qPCR) for a subset of genes (fig. S1G). We therefore assessed the metabolic status of ACC^Δ*LysM*^ macrophages using extracellular flux analysis. We found that BMDMs isolated from ACC^Δ*LysM*^ mice have increased rates of extracellular acidification than control macrophages (Fig. 1F), which corresponded to an increase in both basal glycolytic rate (Fig. 1G) and oligomycin-stressed glycolytic rate (Fig. 1H). Glucose utilization, as assessed by 2NDB-glucose uptake (Fig. 1I) and accumulation of extracellular lactate (Fig. 1J), was also significantly increased in BMDMs isolated from ACC^Δ*LysM*^ mice. On the other hand, analysis of mitochondrial function in ACC^Δ*LysM*^ BMDMs demonstrated a nonsignificant trend to increased basal and maximal oxygen consumption rate (OCR) (fig. S1, H to J). Together, these data demonstrate that loss of ACC, in addition to lipogenesis, affects cellular metabolism, shifting macrophages to a hyperenergetic, hyperglycolytic phenotype at baseline (Fig. 1K), which may affect innate effector functions of macrophages after stimulation.

### Loss of ACC impairs proinflammatory macrophage activation in vitro

**Fig. 2.**
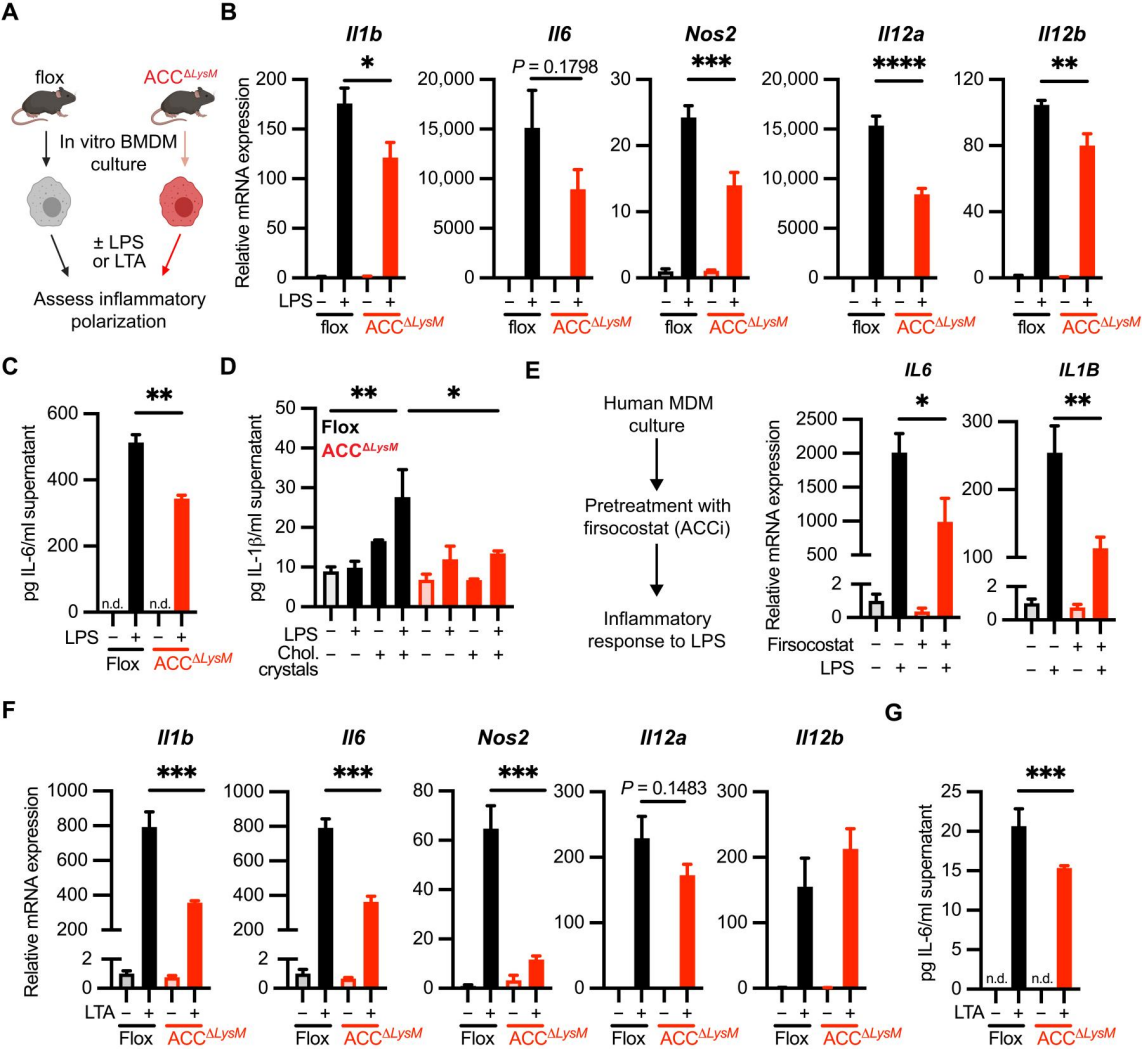
ACC regulates TLR-induced macrophage polarization. (**A**) Schematic of M1 polarization of flox and ACC^Δ*LysM*^ BMDMs. (**B**) Relative mRNA expression of *Il1b*, *Il6*, *Nos2*, *Il12a*, and *Il12b* genes in flox or ACC^Δ*LysM*^ BMDMs stimulated for 6 hours with LPS (100 ng/ml) (*n* = 4). (**C**) Levels of IL-6 protein in supernatant of flox or ACC BMDMs stimulated with LPS for 18 hours (*n* = 4). n.d., not detected. (**D**) Levels of IL-1β protein in supernatant of flox or ACC BMDMs stimulated with LPS for 4 hours followed by cholesterol crystals for 18 hours (*n* = 3 to 4). (**E**) Relative mRNA expression of *IL1B* and *Il6* in human MDMs pretreated with the ACC inhibitor (ACCi) firsocostat (10 μM) before stimulation with LPS (100 ng/ml) for 6 hours (*n* = 4 replicates). (**F**) Relative mRNA expression of *Il1b*, *Il6*, *Nos2*, *Il12a*, and *Il12b* genes in flox or ACC^Δ*LysM*^ BMDMs stimulated for 6 hours with LTA (1 μg/ml) (*n* = 4). (**G**) Levels of IL-6 protein in supernatant of flox or ACC^Δ*LysM*^ BMDMs stimulated with LTA for 18 hours (*n* = 4). Data are represented as means ± SEM. Significance determined by one-way analysis of variance (ANOVA) (B and D to F) or one-tailed Welch’s *t* test (C and G). *P < 0.05, **P < 0.01, ***P < 0.001, and ****P < 0.0001. (A) Created using BioRender.com. See also figs. S2 and S3, related to Fig. 2.

When we used RNA-seq analysis to assess the impact of LPS stimulation on ACC isoform expression, we found that treatment for 6 hours with LPS increased expression of both *Acaca* and *Acacb* (fig. S2A). This was associated with a trend toward increased ACC protein in LPS-treated BMDMs (fig. S2B). We therefore assessed the ability of BMDMs from ACC^Δ*LysM*^ mice to polarize in response to proinflammatory stimuli in vitro (Fig. 2A). We found that, compared to flox controls, induction of the proinflammatory genes *Il6*, *Il1b*, *Nos2*, *Il12a*, and *Il12b* was attenuated in ACC^Δ*LysM*^ BMDMs after treatment with LPS (Fig. 2B). We also observed a small but significant attenuation of LPS-induced expression of the inflammatory surface marker *Cd80*, but not *Cd86*, in response to LPS (fig. S2C). Furthermore, ACC^Δ*LysM*^ BMDMs treated with LPS secreted significantly less IL-6 protein into the supernatant (Fig. 2C). ACC^Δ*LysM*^ BMDMs also demonstrated deficiencies in proteasome-dependent cytokine production, because they released less IL-1β in response to LPS priming followed by NLRP3 activation through exposure to cholesterol crystals (*29*, *30*) (Fig. 2D). To extend our findings to human macrophages, we first assessed the impact of inflammatory stimulation on *ACC* isoform expression in human monocyte-derived macrophages (hMDMs). We observed no changes in *ACACA* or *ACACB* expression (fig. S3A) or ACC protein (fig. S3B) at 6 hours in response to LPS, while at 18 hours LPS induced a significant increase in *ACACB* expression (fig. S3C). We treated hMDMs with the pharmacological ACC inhibitor firsocostat to assess the role of ACC in the LPS response of human macrophages. Compared to vehicle-treated controls, hMDMs treated with firsocostat had significantly decreased expression of *IL6* and *IL1B* after stimulation with LPS for 6 hours (Fig. 2E), suggesting a similar role for ACC in the inflammatory response in both mouse and human macrophages. The blunted inflammatory response in ACC^Δ*LysM*^ BMDMs could also be extended to Gram-positive (TLR2)–dependent activation of the proinflammatory response, as stimulation with lipoteichoic acid (LTA), a TLR2 ligand derived from the Gram-positive bacterium *Staphylococcus aureus*, also demonstrated significantly decreased expression of *Il1b*, *Il6*, and *Nos2* in BMDMs from ACC^Δ*LysM*^ mice (Fig. 2F). We did not observe differences in the induction of surface markers *Cd80* and *Cd86* (fig. S2D). Furthermore, ACC^Δ*LysM*^ macrophages stimulated with LTA secreted significantly lower levels of IL-6 into the supernatant compared with BMDMs from flox controls (Fig. 2G).

To assess whether this defect in inflammatory polarization was due to an alteration in intracellular signaling, we assessed activation of the extracellular signal–regulated kinase (ERK) and nuclear factor κB (NFκB) pathways, which are induced downstream of TLRs and required for the inflammatory response (*31*, *32*). In response to LPS stimulation, BMDMs from ACC^Δ*LysM*^ mice showed similar levels of phosphorylated ERK1/2 at T202/Y204 as flox controls (fig. S2E). This was also the case with phosphorylation of p65 NFκB at the activating S536 (fig. S2F). LPS stimulation also induced similar levels of p65 NFκB nuclear translocation in flox and ACC^Δ*LysM*^ BMDMs (fig. S2G). These data suggest that the blunted inflammatory response in ACC-deficient macrophages was not due to a defect in TLR-activated intracellular signaling.

### ACC-deficient macrophages have an impaired transcriptional response to LPS in vitro

**Fig. 3.**
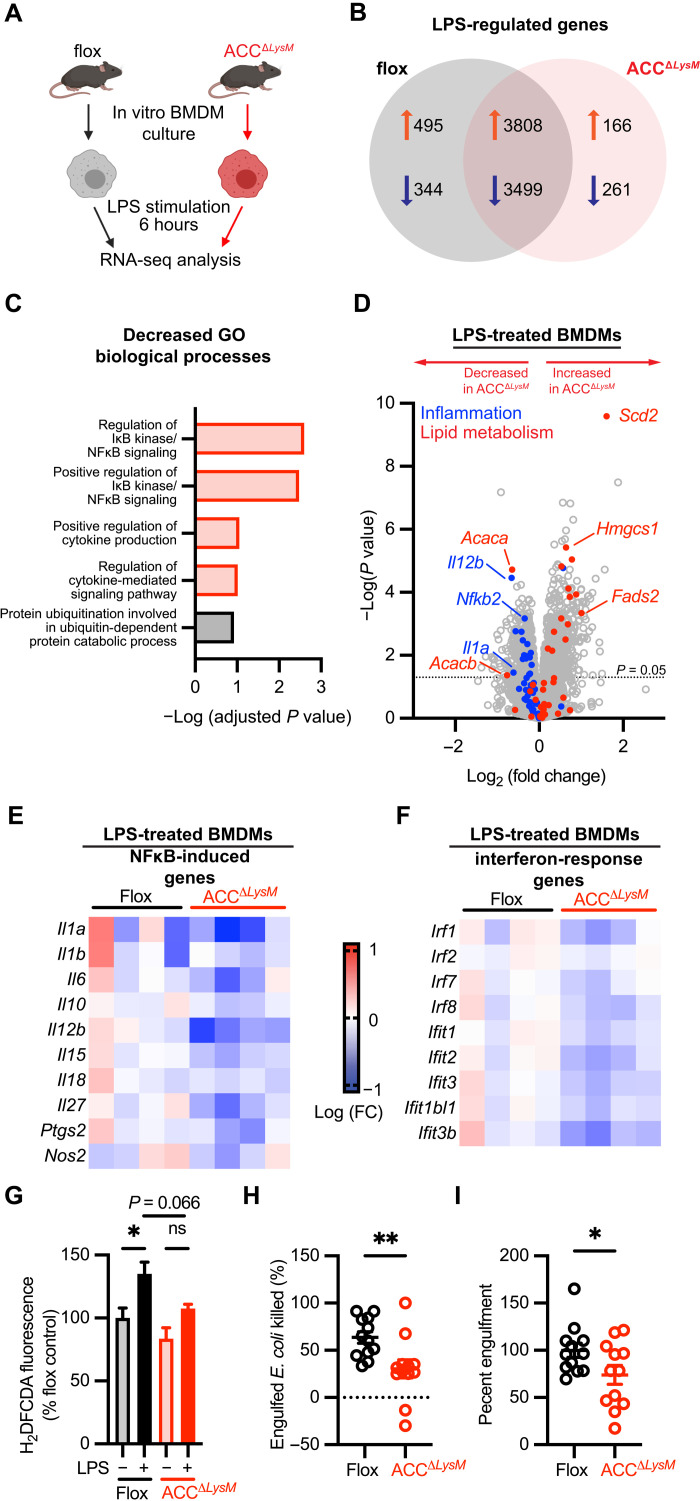
ACC deficiency inhibits LPS-induced macrophage polarization and effector function. (**A**) Schematic showing experimental design of RNA-seq experiment from flox or ACC^Δ*LysM*^ BMDMs at baseline or after stimulation with LPS (100 ng/ml) for 6 hours. (**B**) Venn diagram showing number of genes uniquely regulated in flox or ACC^Δ*LysM*^ BMDMs stimulated with LPS and number of commonly regulated genes. (**C**) GO of genes with decreased expression [−log_2_ (fold change), *P* < 0.05)]. Processes related to inflammation highlighted in red. (**D**) Volcano plot showing relative gene expression of genes in LPS-stimulated ACC^Δ*LysM*^ BMDMs compared with LPS-stimulated flox controls. Blue circles represent genes related to the inflammatory response, and red circles represent lipid metabolism genes. (**E**) Heatmap showing relative expression of NFκB-induced genes in LPS-stimulated flox and ACC^Δ*LysM*^ BMDMs. (**F**) Heatmap showing relative expression of interferon-responsive genes in LPS-stimulated flox and ACC^Δ*LysM*^ BMDMs. (**G**) Relative H_2_DFCDA fluorescence in flox or ACC^Δ*LysM*^ BMDMs stimulated with LPS for 6 hours (*n* = 8). ns, not significant. (**H**) Fraction of engulfed *E. coli* killed by BMDMs 2 hours after engulfment (*n* = 12). (**I**) Percent engulfment of *E. coli* by flox or ACC^Δ*LysM*^ BMDMs after 30 min (*n* = 12). Data are represented as means ± SEM. Significance determined by one-way ANOVA (G) or one-tailed Welch’s *t* test (H and I). *P < 0.05 and **P < 0.01. (A) Created using BioRender.com.

To investigate the mechanisms underlying the attenuation of LPS-induced inflammation, we profiled the transcriptional landscape of control ACC^Δ*LysM*^ BMDMs that were stimulated for 6 hours with LPS using RNA-seq (Fig. 3A). Differential gene expression analysis of LPS-stimulated flox and ACC^Δ*LysM*^ BMDMs identified more than 8000 genes regulated in response to LPS, with 166 uniquely up-regulated and 261 uniquely down-regulated transcripts in ACC^Δ*LysM*^ BMDMs (Fig. 3B). We used the Enrichr gene set enrichment tool (*33*, *34*) to assess the GO biological processes associated with down-regulated genes in LPS-stimulated ACC^Δ*LysM*^ BMDMs compared with LPS-stimulated flox BMDMs. Analysis of the significantly decreased genes identified pathways including “regulation of IκB kinase/NF-κB signaling,” “positive regulation of IκB kinase/NF-κB signaling,” “positive regulation of cytokine production,” and “regulation of cytokine-mediated signaling pathway” among the decreased biological processes (Fig. 3C). Relative to LPS-stimulated flox control cells, BMDMs from ACC^Δ*LysM*^ mice showed an overall decrease in expression of genes associated with the inflammatory response, while genes related to lipid metabolism were significantly increased (*Scd2*, *Fads2*, and *Hmgcs1*) (Fig. 3D). Further analysis revealed a common trend in decreased expression of cytokines and other proinflammatory proteins induced in response to NFκB (Fig. 3E) and interferon-induced genes (Fig. 3F).

We then assessed the functional consequences of the impaired inflammatory response in ACC-deficient BMDMs. Generation of reactive oxygen species (ROS) is critical for pathogen destruction by macrophages (*35*). While LPS stimulation significantly increased levels of ROS in control BMDMs, the accumulation of ROS was blunted in ACC-deficient BMDMs (Fig. 3G). We then used an in vitro bacterial killing assay to assess whether deletion of ACC affects macrophage bactericidal capacity. Compared to control cells, ACC-deficient BMDMs killed a significantly lower percentage of engulfed *Escherichia coli* after 2 hours (Fig. 3H) and demonstrated significant defect in bacterial engulfment (Fig. 3I). Together, these data demonstrate that ACC is a central regulator of the transcriptional and functional aspects of the macrophage immune response.

### ACC is dispensable for in vitro polarization in response to IL-4

**Fig. 4.**
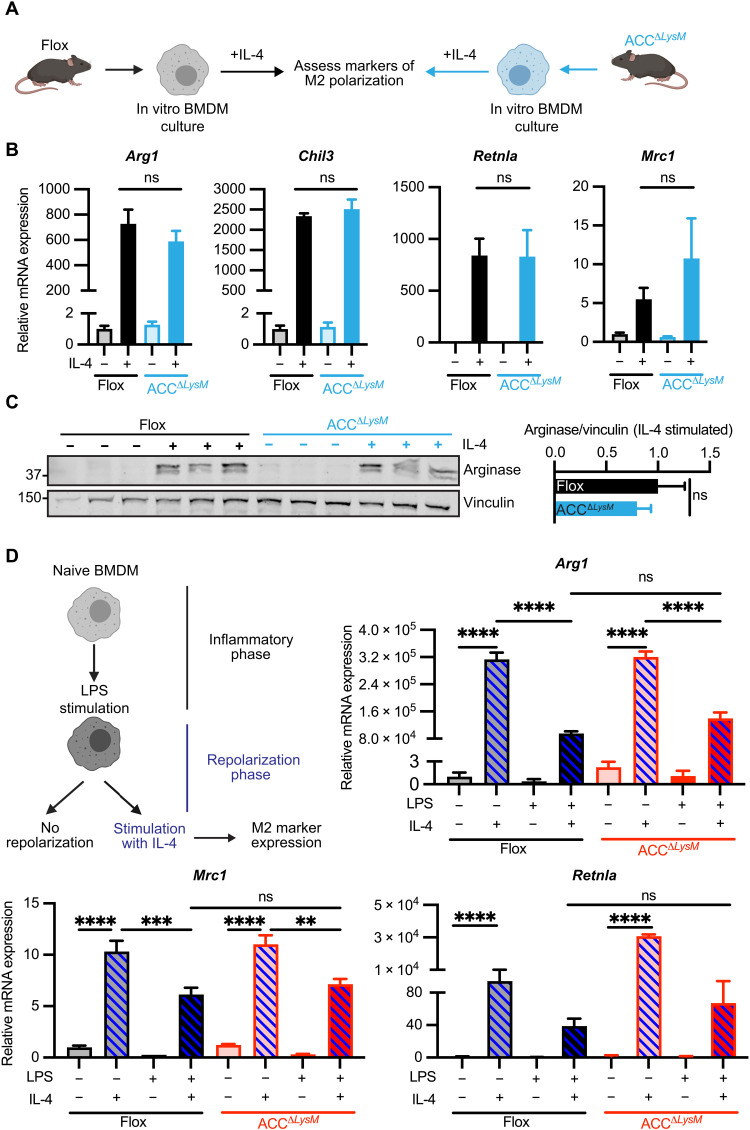
ACC is dispensable for in vitro polarization in response to IL-4. (**A**) Schematic of M2 polarization of flox and ACC^Δ*LysM*^ BMDMs. (**B**) Relative mRNA expression of M2 marker genes *Arg1*, *Chil3*, *Mrc1*, and *Retnla* in flox or ACC^Δ*LysM*^ BMDMs stimulated with vehicle or IL-4 (10 ng/ml) for 6 hours (*n* = 4). (**C**) Whole-cell immunoblot analysis of arginase protein levels in flox or ACC^Δ*LysM*^ BMDMs stimulated with IL-4 (10 ng/ml) for 6 hours (*n* = 3 mice per group). Vinculin was used as a loading control. (**D**) Flox or ACC^Δ*LysM*^ BMDMs were polarized with LPS (100 ng/ml) for 24 hours before repolarization with IL-4 (10 ng/ml) for an additional 24 hours, and expression of *Arg1*, *Mrc1*, and *Retnla* was measured (*n* = 4). Data are represented as means ± SEM. Significance determined by one-way ANOVA (B and D) or one-tailed Welch’s *t* test (C). **P < 0.01. ***P < 0.001, and ****P < 0.0001. (A and D) Created using BioRender.com. See also figs. S3 and S4, related to Fig. 4.

To determine whether ACC plays a role in activation of macrophages to alternative stimuli, we examined IL-4–driven macrophage polarization in ACC^Δ*LysM*^ BMDMs (Fig. 4A). In contrast to LPS stimulation, treatment with IL-4 did not alter the expression of ACC isoforms in either mouse (fig. S4A) or human (fig. S3, A to D) macrophages. In response to IL-4 treatment, ACC^Δ*LysM*^ BMDMs showed no difference in the transcriptional up-regulation of the IL-4–responsive genes *Arg1*, *Chil3*, *Mrc1*, and *Retnla* compared to flox controls (Fig. 4B). This was associated with similar levels of the phosphorylation of signal transducer and activator of transcription 6 (STAT6) at tyrosine-641 and arginase, 6 hours after IL-4 stimulation (Fig. 4C and fig. S4B). The transition from the induction of inflammation to resolution has been proposed to involve phenotypic switching of highly plastic infiltrating macrophages. Therefore, we assessed the ability of M1-polarized ACC^Δ*LysM*^ BMDMs to repolarize in response to subsequent IL-4–induced M2 polarization (Fig. 4D). We found that ACC^Δ*LysM*^ macrophages, which had been previously stimulated with LPS for 24 hours, responded similarly to repolarization with IL-4 with respect to the up-regulation of *Arg1*, *Mrc1*, and *Retnla*, compared to BMDMs isolated from flox controls (Fig. 4D). Together, these data show that decreased ACC expression has neither an impact expression of typical IL-4–responsive genes nor does it affect repolarization from LPS-polarized macrophages, demonstrating that the defect in stimulus-induced gene expression observed in ACC^Δ*LysM*^ macrophages is specific for proinflammatory stimulation.

### ACC is required for the TLR-induced switch to glycolysis in macrophages

The baseline perturbation of macrophage metabolism observed in ACC^Δ*LysM*^ BMDMs prompted us to investigate the impact of ACC knockdown on stimulation-dependent rewiring of macrophage metabolism. TLR stimulation results in a rapid reprogramming of macrophage metabolism, which is required for inflammatory gene transcription, cytokine production, and pathogen destruction (*2*, *3*, *36*). An increase in aerobic glycolysis and a concomitant decrease in oxidative phosphorylation are hallmarks of LPS-polarized macrophages (*3*). Our data show that ACC deficiency shifts macrophages to a hyperglycolytic bioenergetic state (Fig. 1). To assess the glycolytic rate of ACC-deficient BMDMs in response to the TLR4 ligand LPS, we measured extracellular acidification rate (ECAR) for 6 hours after LPS stimulation in control and ACC^Δ*LysM*^ macrophages.

**Fig. 5.**
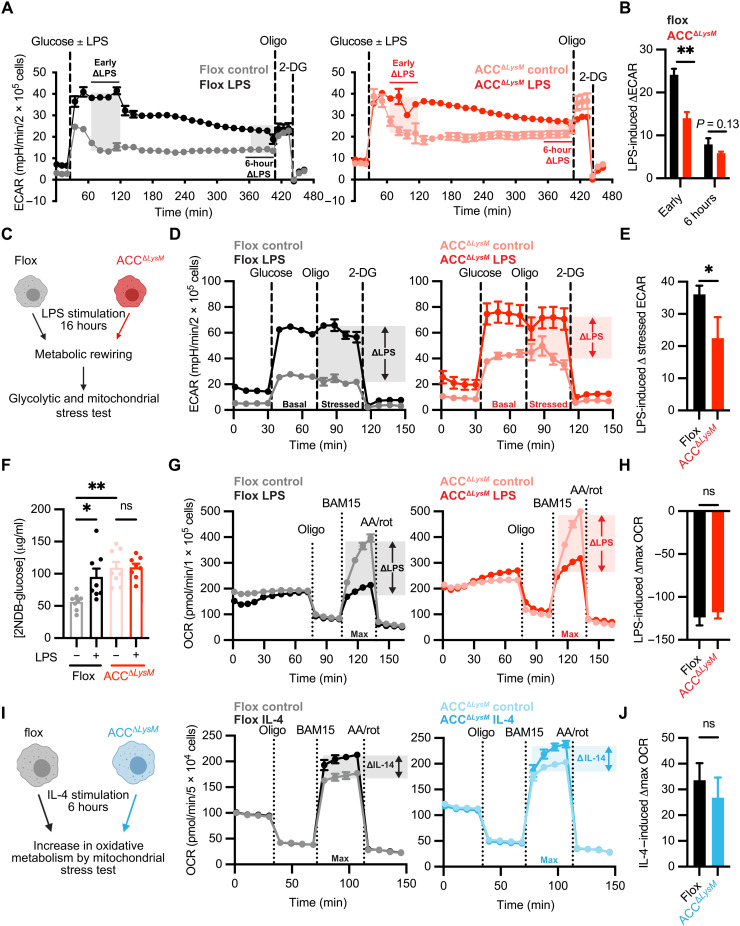
ACC in macrophages regulates the TLR-induced increase in aerobic glycolysis. (**A**) BMDMs from flox or ACC^Δ*LysM*^ mice were stimulated with glucose ± LPS (100 ng/ml), and glycolytic rate was assessed by following ECAR for 6 hours before sequential stimulation with oligomycin and 2-deoxyglucose. The LPS-induced increase in ECAR approximately 30 min after LPS stimulation (early ΔECAR) and approximately 6 hours after LPS stimulation (6 hour ΔECAR) is represented with shaded areas (*n* = 4). (**B**) Quantification of LPS-induced ΔECAR in flox and ACC^Δ*LysM*^ BMDMs (*n* = 4). (**C**) Flox and ACC^Δ*LysM*^ BMDMs were stimulated with LPS for 16 hours before assessment of glycolytic rate, glucose uptake, and mitochondrial function. (**D**) Glycolytic stress test of flox or ACC^Δ*LysM*^ BMDMs after stimulation with LPS (*n* = 5). (**E**) Quantification of LPS-induced change in stressed ECAR in flox or ACC^Δ*LysM*^ BMDMs after 16 hours of stimulation with LPS (*n* = 5). (**F**) 2NDB-glucose uptake in flox or ACC^Δ*LysM*^ BMDMs stimulated with LPS (*n* = 8). (**G**) Flox or ACC^Δ*LysM*^ BMDMs were stimulated with LPS for 16 hours, and OCR was measured (*n* = 5 to 6). (**H**) LPS-induced change in maximal OCR in flox or ACC^Δ*LysM*^ BMDMs (*n* = 5 to 6). (**I**) Flox or ACC^Δ*LysM*^ BMDMs were stimulated with IL-4 for 6 hours before mitochondrial stress test (*n* = 6). (**J**) Quantification of IL-4–induced change in maximal OCR (*n* = 6). Data represented as means ± SEM. Significance determined by one-tailed Welch’s *t* test (B, E, H, and J) or one-way ANOVA (F). *P < 0.05 and **P < 0.01. (C and I) Created using BioRender.com. See also fig. S5, related to Fig. 5.

Stimulation with LPS resulted in an increase in glycolytic rate within the first hour after treatment in BMDMs from flox control mice (early ΔLPS; Fig. 5A, left). However, the early LPS-dependent increase in glycolytic rate was less pronounced in ACC-deficient BMDMs (early ΔLPS; Fig. 5A, right), indicated by lower early LPS-induced change in ECAR compared with flox controls (Fig. 5B). Differences were less pronounced at 6 hours. To assess whether this defect in LPS-induced metabolic polarization was specific to the early increase in glycolysis, we polarized macrophages with LPS for 16 hours, a time point at which LPS induces suppression of mitochondrial oxidative phosphorylation, and assessed glycolytic rate, glucose uptake, and mitochondrial function (Fig. 5C). Glycolytic stress tests of BMDMs after 16 hours of stimulation with LPS revealed a significantly lower increase in stressed ECAR in ACC-deficient BMDMs (Fig. 5, D and E), and a similar response was seen after 16 hours of stimulation with the TLR2 ligand LTA (fig. S5, A and B). Furthermore, while stimulation with LPS increased glucose uptake in control BMDMs, there was no further increase in glucose uptake in response to LPS in ACC-deficient macrophages (Fig. 5F). Assessing the mechanism behind the differences in basal and LPS-induced glycolysis, we found that ACC^Δ*LysM*^ BMDMs had increased levels of cell surface–associated GLUT1 compared to flox controls, as assessed by cell surface biotinylation assay (fig. S5, C and D). However, while LPS increased the levels of GLUT1 associated with the cell surface in control macrophages, this response was not evident in ACC^Δ*LysM*^ BMDMs stimulated with LPS (fig. S5, C and D). Together, these data suggest that by increasing the baseline rate of glucose uptake, decreased levels of ACC in macrophages set an upper limit of glycolytic rate in response to LPS.

We then asked whether ACC played a role in other aspects of LPS-induced metabolic rewiring. Succinate dehydrogenase (SDH) activity, which has been reported to increase in response to LPS (*37*), was similar between control and ACC^Δ*LysM*^ BMDMs under both basal and LPS-stimulated conditions (fig. S5E). We also saw similar levels of reduced (fig. S5F) and oxidized NADPH (fig. S5G) and a similar increased NADP^+^/NADPH ratio (fig. S5H) in response to LPS in control and ACC^Δ*LysM*^ BMDMs. We also assessed the impact of ACC knockdown on LPS-induced decrease in mitochondrial oxygen consumption by mitochondrial stress test (Fig. 5G). Compared to flox controls, BMDMs from ACC^Δ*LysM*^ mice demonstrated a similar decrease in maximal OCR in response to LPS treatment (Fig. 5H), suggesting that ACC is critical for the LPS-dependent switch to aerobic glycolysis, but not the later suppression of mitochondrial function. Transcriptional analysis of genes encoding tricarboxylic acid (TCA) cycle–related enzymes revealed modest increase in levels of *Idh1* (encoding isocitrate dehydrogenase) and *Pck2* (encoding mitochondrial phosphoenolpyruvate carboxykinase) 6 hours after LPS stimulation in BMDMs with decreased levels of ACC (fig. S5I), but overall expression levels of TCA enzymes were similar between control and ACC^Δ*LysM*^ BMDMs. Stimulation with LPS or LTA also induced increases in extracellular lactate levels in both flox and ACC^Δ*LysM*^ BMDMs (fig. S5J).

Stimulus-dependent metabolic rewiring is essential not only for proinflammatory polarization but also for alternative activation by IL-4 (*4*); therefore, we assessed whether loss of ACC plays a role in the increase of mitochondrial oxygen consumption in response to IL-4 stimulation (Fig. 5I). In both flox and ACC^Δ*LysM*^ BMDMs, treatment with IL-4 resulted in an increase in maximum OCR (Fig. 5J), suggesting that loss of ACC does not impair the bioenergetic changes required for IL-4–dependent activation. Together, these data demonstrate that loss of ACC specifically impairs the early rewiring of glucose metabolism in response to proinflammatory stimulation, highlighting an unexpected role for ACC as a central regulator of metabolic control of macrophage inflammation.

### ACC is required for LPS-induced modification of the macrophage lipidome

Activation of the de novo lipogenesis and cholesterol biosynthesis pathways is crucial for the inflammatory response to LPS (*8*, *9*, *38*, *39*). Given the role of ACC in lipid synthesis and based on our observation that loss of ACC blunts LPS-induced switch to glycolytic metabolism, we tested whether ACC is required for LPS-induced redistribution of lipid metabolism as well.

**Fig. 6.**
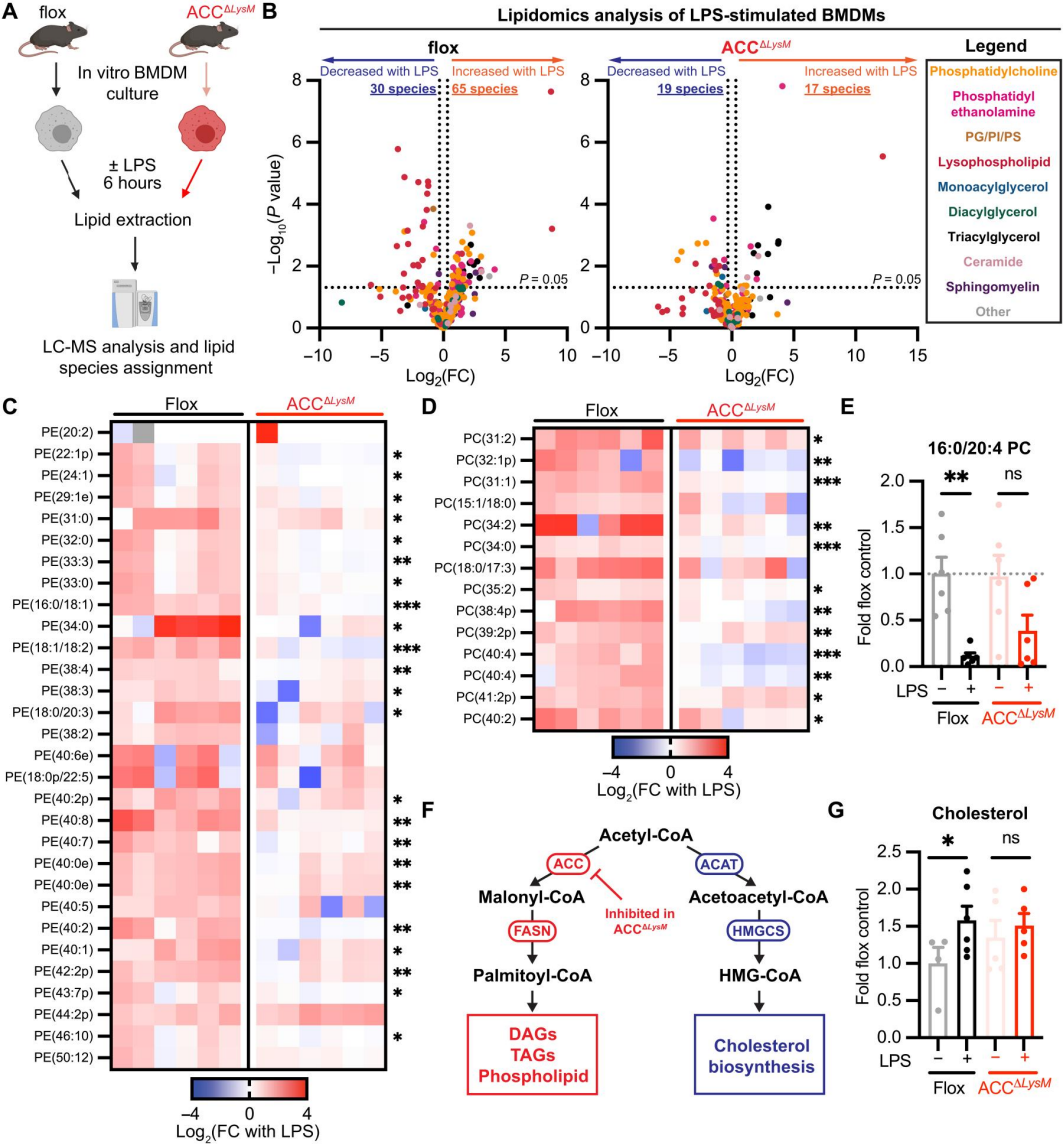
ACC is required for LPS-induced rewiring of the macrophage lipidome. (**A**) Schematic of lipidomic analysis workflow from flox or ACC^Δ*LysM*^ BMDMs stimulated with LPS (100 ng/ml) for 6 hours. (**B**) Volcano plots representing fold change of lipid species abundance in flox or ACC^Δ*LysM*^ BMDMs stimulated with LPS (100 ng/ml) for 6 hours (*n* = 6). (**C**) Heatmap of significantly increased phosphatidylethanolamine (PE) species in LPS-stimulated flox and ACC^Δ*LysM*^ BMDMs, relative to respective unstimulated cells (*n* = 6). (**D**) Heatmap of significantly increased phosphatidylcholine (PC) species in LPS-stimulated flox and ACC^Δ*LysM*^ BMDMs relative to respective unstimulated cells (*n* = 6). (**E**) Levels of 16:0/20:4 phosphatidylcholine in flox or ACC^Δ*LysM*^ BMDMs stimulated with LPS (100 ng/ml) for 6 hours (*n* = 6). (**F**) Schematic showing fates of acetyl-CoA in the de novo lipogenesis and sterol biosynthesis pathways. (**G**) Levels of cholesterol in flox or ACC^Δ*LysM*^ BMDMs stimulated with LPS (100 ng/ml) for 6 hours (*n* = 4 to 6). Data are represented as means ± SEM (E and G). Significance determined by two-tailed Student’s *t* test (C and D) or one-way ANOVA (E and G). *P < 0.05, **P < 0.01, and ***P < 0.001. (A) Created using BioRender.com. See also fig. S6, related to Fig. 6.

Using an unbiased liquid chromatography–mass spectrometry (LC-MS)–based lipidomics approach, we assessed the impact of ACC deficiency on macrophage lipid metabolism in response to LPS stimulation (Fig. 6A). We examined the role for ACC in LPS-induced modulation of lipid metabolism in the context of full culture medium supplementation, mirroring the conditions of our bioenergetics analyses. In comparison to unstimulated cells, LPS stimulation of BMDMs from control mice for 6 hours resulted in redistribution of lipid species across lipid classes (Fig. 6B, left), with 65 lipid species increased in abundance and 30 species decreased in abundance after LPS stimulation (|fold change| > 1.25; *P* < 0.05), representing changes in approximately 23% of total detected species (420). Strikingly, in comparison to control cells, LPS treatment resulted in regulation of only 36 total species (17 increased and 19 decreased) in ACC^Δ*LysM*^ BMDMs (Fig. 6B, right). Examination of the 65 species increased by LPS in control macrophages revealed that LPS-driven enrichment of phosphatidylcholine and phosphatidylethanolamine species was attenuated in ACC-deficient BMDMs (Fig. 6, C and D). We also observed attenuated accumulation of diacylglycerol, sphingomyelin, and ceramide species in ACC-deficient BMDMs treated with LPS (fig. S6, E and F).

LPS also stimulates the release of arachidonic acid from arachidonoyl-containing phospholipids by phospholipases, which is then converted to proinflammatory eicosanoids. Levels of the arachidonoyl-containing phospholipid 1-palmitoyl-2-arachidonoyl-*sn*-glycero-3-phosphocholine (16:0/20:4, PC/PAPC) were significantly decreased in control macrophages after LPS stimulation, while in ACC-deficient macrophages, levels of PAPC trended lower in response to LPS but did not reach statistical significance (Fig. 6E). We then considered how loss of ACC might affect other lipid metabolism–related processes stimulated by LPS. Acetyl-CoA is a common substrate for both de novo lipogenesis and cholesterol biosynthesis (Fig. 6F), and because expression levels of enzymes in the sterol biosynthesis pathway were increased in LPS-stimulated ACC^Δ*LysM*^ BMDMs (fig. S4, B and C), we assessed free cholesterol levels by LC-MS. Unstimulated ACC-deficient BMDMs showed a trend toward an increase in cholesterol levels compared with flox controls (Fig. 6G). However, while LPS treatment significantly increased cholesterol levels in control macrophages, we observed no changes in cholesterol levels in ACC^Δ*LysM*^ BMDMs upon LPS stimulation (Fig. 6G).

Transcriptional analysis using RNA-seq revealed that induction of genes associated with the de novo lipogenesis and cholesterol biosynthesis pathways was even more pronounced in LPS-stimulated ACC^Δ*LysM*^ BMDMs compared to flox controls (fig. S6, A and B). This was associated with slightly increased baseline expression levels of *Srebf1* and *Srebf2*, transcription factors that control lipid metabolism (fig. S6C). *Nr1h3* (encoding LXRα) and *Nr1h2* (encoding LXRβ) expression were similar between genotypes at baseline and similarly induced after LPS treatment (fig. S6C). We confirmed induction of genes involved in cholesterol synthesis by qPCR (fig. S6D). When we assessed accumulation of ^14^C-labeled acetate into the lipid-soluble fraction of flox and ACC^Δ*LysM*^ macrophages, we observed a pattern suggestive of lower incorporation of labeled acetate in ACC^Δ*LysM*^ macrophages under control conditions after 6 hours (fig. S6G). However, when we first stimulated BMDMs with LPS for 4 hours before exposing them to ^14^C-acetate for a 2-hour period, we found that the fold increase in ^14^C-acetate incorporation rate induced by LPS was blunted in ACC^Δ*LysM*^ macrophages (fig. S6H). Both sterols and fatty acid–containing lipids partitioned into the lipid-soluble layer and can be made from acetate (Fig. 6F). Because ACC^Δ*LysM*^ macrophages demonstrate an up-regulation of genes involved in cholesterol synthesis (fig. S6, B and D), it is possible that, given that we observed a trend toward increased cholesterol sterol levels in ACC^Δ*LysM*^ macrophages (Fig. 6G), the effect of decreasing ACC levels is partially masked using this method, where all lipid-soluble species are measured together.

Together, these data reveal that rapid rewiring of the macrophage lipidome that occurs within 6 hours in response to LPS is dependent on ACC. Macrophages with decreased levels of ACC demonstrate profound deficiencies in lipidomic reprogramming, compensated by increased expression of lipogenic genes, which is critical for this component of the proinflammatory metabolic response.

### Genetic or pharmacologic manipulation of ACC1 and ACC2 attenuates LPS-induced peritoneal inflammation but exacerbates bacterial peritonitis in mice

**Fig. 7.**
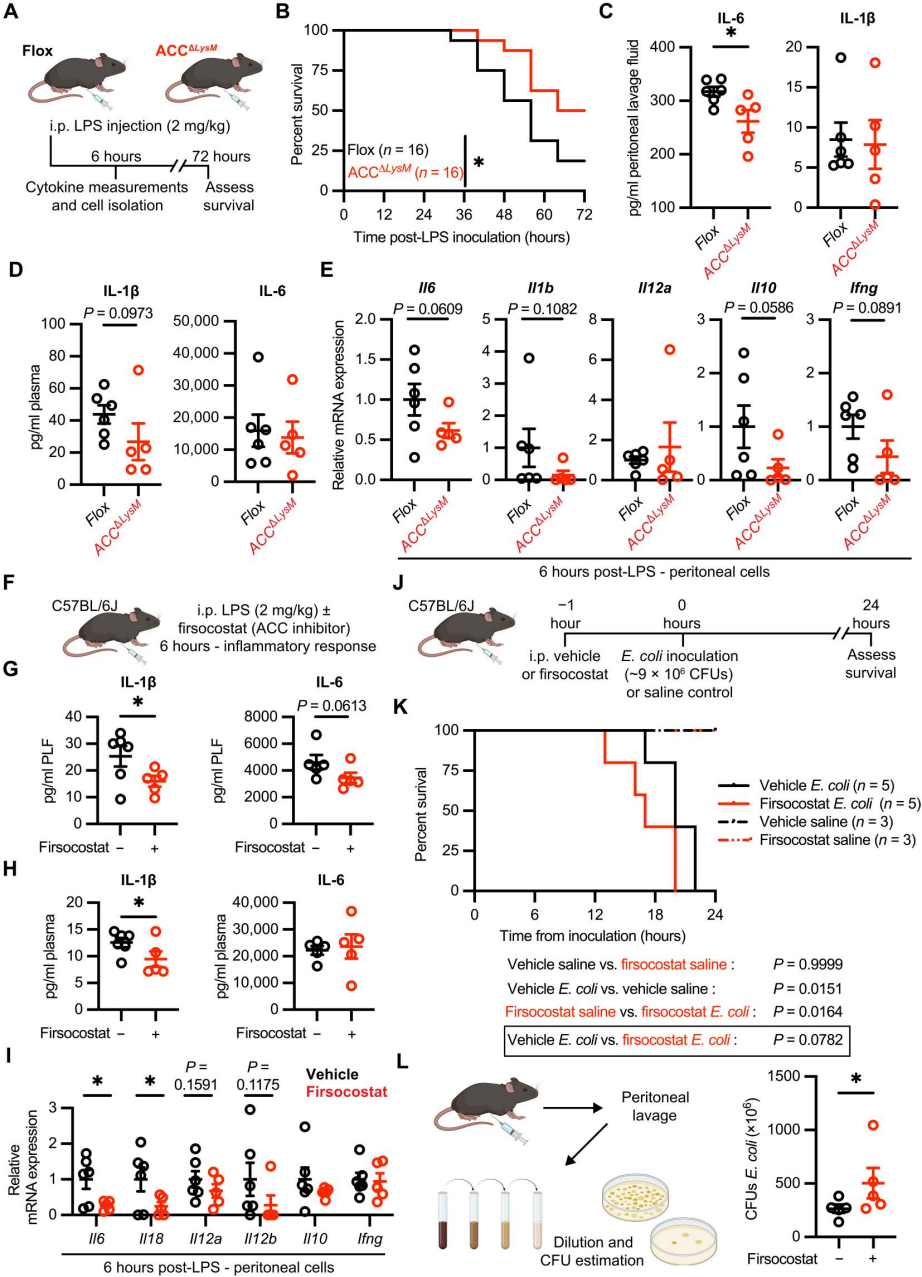
Genetic or pharmacologic manipulation of ACC1 and ACC2 attenuates LPS-induced peritoneal inflammation but exacerbates bacterial peritonitis in mice. (**A**) Experimental schematic of LPS-induced model of systemic inflammation. (**B**) Survival curves of male flox or ACC^Δ*LysM*^ mice after intraperitoneal LPS administration (*n* = 16). (**C**) Peritoneal lavage cytokine levels 6 hours after LPS administration (*n* = 5 to 6). (**D**) Plasma cytokine levels 6 hours after LPS administration (*n* = 5 to 6). (**E**) Relative mRNA levels in peritoneal cells from flox or ACC^Δ*LysM*^ mice 6 hours after LPS (*n* = 5 to 6). (**F**) C57BL/6J mice were injected with LPS (2 mg/kg) and either DMSO control or firsocostat (25 mg/kg), and cytokines and peritoneal cell mRNA expression were measured at 6 hours. (**G**) Peritoneal lavage cytokine levels 6 hours after LPS (*n* = 5 to 6). (**H**) Plasma cytokine levels 6 hours after LPS (*n* = 5 to 6). (**I**) Relative mRNA levels in peritoneal cells isolated 6 hours after LPS administration (*n* = 5 to 6). For (G) to (I), one outlier in the firsocostat treatment group was removed (ROUT *Q* = 1%). (**J**) C57BL/6J mice were pretreated with firsocostat (25 mg/kg) before *E. coli* inoculation [~9 × 10^6^ colony-forming units (CFUs) per mouse] or saline. (**K**) Survival of mice pretreated with control or firsocostat before *E. coli* inoculation or saline (saline, *n* = 3; *E. coli*, *n* = 5). (**L**) Peritoneal lavage of inoculated mice was serially diluted, and bacterial CFUs were estimated (*n* = 5). Data represented as means ± SEM. Significance determined by one-tailed Welch’s *t* test (C to E and G to I), Mantel-Cox test (B and K), or one-tailed Mann-Whitney test (L). *P < 0.05 and **P < 0.01. (A, F, J, and L) Created using BioRender.com.

We then sought to examine whether decreased ACC affected the inflammatory response in vivo. To this end, we subjected mice to a model of LPS-induced systemic inflammation (*40*), in which onset of symptoms occurs between 6 and 24 hours and persists for 48 to 72 hours after LPS administration (Fig. 7A). ACC^Δ*LysM*^ mice responded significantly better to LPS-induced peritonitis, with an increased median survival time (68 hours), compared with flox control (56 hours) (Fig. 7B). The improved survival in response to LPS was associated with significantly lower levels of the proinflammatory cytokines IL-6 in the peritoneal lavage fluid (Fig. 7C) and IL-1β in the plasma (Fig. 6D) in ACC^Δ*LysM*^ mice 6 hours after LPS administration. Furthermore, analysis of mRNA of peritoneal exudate cells isolated 6 hours after LPS administration revealed trends of lower expression in proinflammatory genes *Il6*, *Il1b*, *Il12a*, *Il10*, and *Ifng* in cells isolated from ACC^Δ*LysM*^ mice compared with flox controls (Fig. 7E).

Pharmacological inhibitors of ACC are in clinical development for metabolic disorders including nonalcoholic steatohepatitis and several cancers (*41*–*43*). Given that ACC^Δ*LysM*^ mice showed an attenuated inflammatory response 6 hours after induction of LPS-induced peritonitis, we examined whether pharmacological inhibition of ACC would also blunt the response to LPS. To this end, we treated mice with the ACC inhibitor firsocostat (*41*) simultaneously with the administration of LPS (Fig. 7F). Mice treated with firsocostat had significantly lower levels of IL-6 and IL-1β in the peritoneal lavage fluid (Fig. 7G) and lower levels of IL-1β in the plasma (Fig. 7H) at 6 hours after LPS administration compared with mice that received vehicle treatment. Furthermore, gene expression analysis of peritoneal exudate cells 6 hours after LPS injection revealed that transcript levels of cytokines including *Il6*, *Il18*, *Il12a*, and *Il12b* were significantly decreased or trending toward decreased in mice treated with firsocostat (Fig. 6I). We then assessed the impact of ACC inhibition in a clinically relevant model of bacterial peritonitis, where both activation of the inflammatory response and control of pathogen growth affect outcomes. We pretreated wild-type mice with firsocostat for 1 hour before intraperitoneal inoculation with *E. coli* O18ac:K1:H7 [approximately 9 × 10^6^ colony-forming units (CFUs) per mouse] or saline control and assessed survival (Fig. 7J). In the *E. coli* treatment group, mice treated with firsocostat had a shorter median survival time (17 hours) compared to controls (20 hours) (Fig. 7K). When we analyzed the bacterial burden in the peritoneal cavities of inoculated mice, we found that mice treated with firsocostat had significantly higher levels of *E. coli* in the peritoneal lavage compared to vehicle controls (Fig. 7L). Together, these data demonstrate that myeloid-specific deficiency or pharmacologic inhibition of ACC blunts the inflammatory response to LPS and innate immune functions of macrophages in vivo.

## DISCUSSION

In response to insults such as infection or tissue damage, immune cells including macrophages must rapidly adapt their intracellular metabolism to mount an effective inflammatory response (*1*, *44*, *45*). Regulation of lipid metabolism is essential for both induction and resolution of the inflammatory response (*9*, *12*, *38*). A role for ACC, the committed step of de novo lipogenesis, has been implicated in regulation of some immune cell subtypes (*26*, *46*, *47*), but its role in myeloid cells remains unexplored. We generated a mouse model with a myeloid-specific knockdown of both ACC1 and ACC2 (ACC^Δ*LysM*^) and used transcriptional, functional, bioenergetics, and lipidomics approaches to assess the impact of decreased ACC levels on macrophage activation. We found that genetic knockdown or pharmacological inhibition of ACC attenuates LPS-induced inflammation in vivo, with reduced levels of proinflammatory cytokines 6 hours after induction of inflammation.

Our data show that decreased levels of ACC switch macrophages to a hyperglycolytic metabolism, with increased basal ECAR and glucose uptake. This mirrors the phenotype of isolated hepatocytes from liver-specific ACC1/ACC2 knockout mice, which demonstrated increased rates of glucose oxidation (*25*), and suggests a cell type–independent effect of ACC knockdown on regulation of glycolysis. Mechanistically, we identified a baseline increase in GLUT1 at the cell surface in ACC^Δ*LysM*^ macrophages, which, unlike flox controls, is not further increased upon LPS stimulation. In other cell types, GLUT1 trafficking to the plasma membrane has been shown to be related to cellular lipid metabolism. In adipocytes, glucose consumption via GLUT1 is related to association of GLUT1 with lipid rafts (*48*), which is enhanced in settings of glucose deprivation. In endothelial cells, the activity of vascular endothelial growth factor–B (VEGF-B) decreases cholesterol localization to the plasma membrane, which impairs GLUT1-dependent glucose metabolism (*49*). The importance of cholesterol for GLUT1 activity at the cell surface has also been demonstrated in multiple human cell types, as decreasing cholesterol levels by statin treatment decreased GLUT1 membrane localization (*50*, *51*). Here, we show that ACC knockdown in macrophages increases expression of cholesterol biosynthesis genes, with higher basal levels of cholesterol, which is not further increased in response to LPS stimulation, which may explain both the basal increase in GLUT1 membrane localization and the differences in glucose uptake and glycolytic rate in response to LPS.

We also observed baseline up-regulation of lipid synthesis pathway genes in ACC-deficient macrophages, demonstrating that the deletion of ACC upsets the baseline metabolic set point of macrophages. As discussed above, our findings of both increased basal glycolytic rate while reaching the same maximal glycolytic rate suggest that there is limited metabolic flexibility in macrophages with decreased levels of ACC. Because flexibility to increase glycolytic flux is essential for polarization of macrophages in response to inflammatory stimuli, the reduced flexibility of ACC^Δ*LysM*^ macrophages may be one contributing factor to the blunted inflammatory response we observed. However, other alterations in metabolism induced by LPS, such as suppression of mitochondrial oxygen consumption, changes in TCA cycle–associated gene expression, and increased SDH activity, were unaffected by ACC knockdown. Together, these findings demonstrate that, by shifting the bioenergetic set point to a hyperglycolytic state, decreased levels of ACC reduce the capacity of macrophages to enhance glycolytic rate in response to TLR activation, which results in attenuation of the inflammatory response.

Stimulus-induced metabolic reprogramming in macrophages is not limited to proinflammatory polarization: Enhancement of lipolysis and mitochondrial oxygen consumption is required for alternative “M2” activation (*4*, *52*), while polarization of macrophages to the redox-responsive “Mox” phenotype reroutes glucose consumption into the pentose phosphate pathway for NADPH production (*6*, *7*). However, we found that the IL-4–induced metabolic and transcriptional responses we evaluated were largely unaffected by ACC deletion, demonstrating that ACC deficiency impairs signal-specific macrophage polarization through selective perturbations in cellular metabolism.

In vitro, BMDMs from ACC^Δ*LysM*^ mice demonstrated an attenuated transcriptional response to polarization with inflammatory stimuli, which resulted in decreased secretion of proinflammatory cytokines and impaired proinflammatory effector function, including ROS production, bacterial engulfment, and pathogen killing. Recent work has demonstrated that the early LPS-stimulated induction of glycolysis activates ACLY, which uses citrate to generate acetyl-CoA for histone acetylation (*36*). The pharmacological inhibition of ACLY attenuated the LPS-induced inflammatory response in vitro and in an in vivo model of LPS endotoxemia (*36*); conversely, myeloid-specific deletion of ACLY resulted in a hyperresponsiveness to LPS in vitro (*28*) but had minimal impact in models of acute or chronic inflammation (*53*). We demonstrate here that either genetic deficiency or pharmacological inhibition of ACC attenuates LPS-induced inflammation in vivo.

Macrophages from ACC^Δ*LysM*^ mice demonstrated a profound defect in reconfiguration of cellular lipid metabolism in response to LPS stimulation. Together with the observation that ACC^Δ*LysM*^ BMDMs demonstrate attenuated expression of proinflammatory cytokines, our findings support previous studies that have demonstrated the link between control of lipid metabolism in macrophages and the inflammatory response (*9*, *11*). In addition, recent work demonstrated that *Drosophila* reroute lipid metabolism toward phospholipid synthesis in response to bacterial infection or genetic activation of the Toll signaling pathway, which facilitates ER expansion and antimicrobial peptide secretion (*54*). Furthermore, SREBP-dependent lipid synthesis is required for macrophage phagocytosis after TLR4 activation (*55*), and here, we find that ACC^Δ*LysM*^ macrophages have both blunted phospholipid accumulation in response to LPS and an attenuated capacity for bacterial engulfment.

Given its key role in the de novo synthesis of fatty acids, inhibition of ACC has become an attractive pharmacological target for nonalcoholic fatty liver disease (*41*, *42*, *56*, *57*). Furthermore, ACC inhibitors are under investigation for treatment of solid malignancies including hepatocellular carcinoma (*58*) and non–small cell lung cancer (*43*). Our findings that (i) treatment with an ACC inhibitor attenuates inflammatory responses in vivo and (ii) macrophages with decreased levels of ACC have a defect in pathogen killing in vitro suggest that attenuation of the immune response is a consequence of inhibition of ACC activity. Furthermore, our observation that treatment with a pharmacological inhibitor of ACC worsened outcomes in a model of peritoneal infection identified a role for ACC in in vivo pathogen control. The role of ACC in control of macrophage function that we identified could potentially be harnessed therapeutically in cases of hyperinflammation, such as seen in patients with sepsis. However, our findings also point toward decreased bacterial killing as a potential unwanted side effect of pharmacological ACC inhibition in the context of metabolic disease or cancer. Previous studies in patients with sepsis have long demonstrated an impact of whole-body lipid metabolism on recovery outcomes (*59*, *60*). Recent studies have shown that metabolic processes including “fatty acid biosynthesis” are associated with nonresponse to therapy in sepsis (*61*), while metabolomics analysis identified a key role for lipid metabolites in stratifying outcomes in septic shock patients (*62*). Furthermore, expression of the lipid-responsive transcription actor peroxisome proliferator–activated receptor α (PPARα) is decreased in patients with sepsis, and decreased PPARα is associated with increased bacterial burden and mortality in a rodent sepsis model (*63*). Our findings further elucidate the role of de novo lipid synthesis in both the hyperinflammatory and bacterial clearance components of systemic infection.

In our genetic model of ACC deficiency, we observed an incomplete deletion of ACC isoforms in our in vitro macrophage culture; nevertheless, we observed significant perturbations in glucose and lipid metabolism at baseline and response to LPS in macrophages with decreased levels of ACC. The partial knockdown of ACC that we observed in our model corresponded to a partial decrease in carbon flux through lipid synthesis pathways, to which both de novo lipogenesis (which ACC knockdown would partially impair) and cholesterol biosynthesis (which is up-regulated in our model) can contribute, which may mask some of the effect in this assay. Nevertheless, we still identified major deficits in LPS-induced increases in multiple lipid species, in particular phospholipid species that contribute to key macrophage functions, including cytokine secretion and bacterial killing, which were impaired in ACC^Δ*LysM*^ macrophages. In this study, we limited most of our metabolic and lipidomic analysis to the first 6 hours after LPS stimulation. Multiple studies have demonstrated that LPS-induced alterations in lipid metabolism evolve during the course of the inflammatory response (*8*, *9*, *13*). Our observation that knockdown of ACC results in a basal increase in glycolysis through both changes in glycolytic gene expression and cell surface association of GLUT1 identifies a novel link between perturbation of the de novo lipogenesis pathway and the metabolic component of inflammatory macrophage polarization. Future studies might evaluate the role of ACC in other aspects of macrophage metabolic responses during distinct stages of initiation and resolution of inflammation. We observed a pattern of decreased gene expression of key inflammatory mediators including *Il1b*, *Il6*, *Il12*, and *Il10*, as well as changes in IL-6 and IL-1β levels, 6 hours after induction of inflammation with LPS in vivo. While we focused our analyses to the initial response to inflammation, future studies analyzing the impact of ACC knockdown at later stages of infection and pharmacological ACC inhibition after the initial inflammatory phase would serve to further elucidate the role of this metabolic regulator during all aspects of the induction and resolution of the inflammatory response.

In conclusion, we identified an unexpected role for ACC in linking metabolic reprogramming and activation of inflammatory responses in macrophages. Using genetic and pharmacological approaches in vivo, as well as bioenergetics, lipidomics, and transcriptional methods in vitro, we found that loss of ACC shifts macrophages to a hyperglycolytic metabolism while limiting the capacity to enhance glucose utilization and blocking lipid accumulation in response to TLR stimulation. ACC knockdown blunted proinflammatory gene transcription, which impaired macrophage cytokine secretion, phagocytosis, and pathogen destruction. These findings identify a role for ACC in regulation of the inflammatory response and underscore the links between control of cellular metabolism and stimulus-dependent macrophage polarization.

## METHODS

### Experimental design

The purpose of this study was to assess the role of ACC in the macrophage acute inflammatory response and the impact of ACC deficiency on macrophage polarization in vitro and the inflammatory response in vivo. For in vitro experiments, BMDMs from age- and sex-matched flox control and *ACC*^Δ*LysM*^ mice were cultured as described below and used for experiments. In vitro experiments were performed using biological replicates of primary cells derived from multiple mice or repeated at least twice, with representative results (technical replicates) presented. Experiments in hMDMs were performed in technical triplicate or quadruplicate as listed in figure legends. In vivo experiments for the LPS-induced peritonitis model used age- and sex-matched flox control and *ACC*^Δ*LysM*^ mice, and for survival experiments, experimenters assessing the clinical score and overall survival were blinded to genotype before the end of the experiment. Sample sizes for in vivo experiments were based on previous publications from our group using this model of LPS-induced endotoxemia (*40*, *64*), and animals from multiple independent litters were used for experiments. Mice were randomized by weight to treatment groups for experiments using wild-type mice where appropriate. Humane end points for in vivo experiments were predefined as described below and approved by the University of Virginia Animal Care and Use Committee. No other components were prespecified.

### Mice

C57BL/6J mice (stock 000664) were purchased from The Jackson Laboratory (Bar Harbor, ME). Myeloid-specific ACC1/ACC2-deficient mice were generated by crossing *Acaca^loxP/loxP^ Acacb^loxP/loxP^* (ACC1/ACC2 floxed) mice (*25*) with B6.129P2-*Lyz2^tm1(cre)Ifo^*/J (LysM Cre) mice (*27*) obtained from The Jackson Laboratory (stock 004781). Mice were backcrossed to give mice homozygous for the *Acaca* and *Acacb* floxed alleles with the LysM Cre. For breeding, mice hemizygous for the LysM Cre transgene were crossed with *Acaca/Acacb* floxed mice to give littermates of *Acaca^loxP/loxP^ Acacb^loxP/loxP^ LysM^wt/wt^* (flox) and *Acaca^loxP/loxP^ Acacb^loxP/loxP^ LysM^cre/wt^* (*ACC*^Δ*LysM*^) mice. Unless otherwise stated, animals were maintained in the University of Virginia Center for Comparative Medicine in a pathogen-free animal facility with ad libitum access to food (standard rodent chow, Teklad) and water and a 12-hour light/12-hour dark cycle. All animal experiments were approved by the University of Virginia Animal Care and Use Committee (protocol #3444). The genomic DNA was isolated from tail clip biopsies by proteinase K (Bioline) digestion in DirectPCR tail lysis reagent (Viagen Biotech). Genotype was confirmed by PCR analysis of genomic DNA using Apex Taq Master Mix (Genesee Scientific) with primers as previously described. Age- and sex-matched littermate controls were used for experiments.

### Endotoxemia model

Ten- to 14-week-old male mice were injected with LPS (2 mg/kg; Ultrapure LPS from *E. coli*, InvivoGen) in sterile saline by intraperitoneal injection, and body mass and rectal temperature were recorded by an observer blinded to the experimental conditions. For ACC inhibitor administration, dimethyl sulfoxide (DMSO) vehicle or firsocostat (Selleck Chemicals) was administered via intraperitoneal injection just before administration of LPS. Clinical symptoms were assessed using a scoring scale as previously described (*64*). At the times indicated, mice were euthanized by carbon dioxide inhalation and cardiac puncture, and lavage and tissues were collected for further analysis. For survival analysis, mice were monitored for weight loss, symptom severity, and survival every 8 hours for 72 hours. Mice found dead/moribund or reaching predetermined humane end points were euthanized.

### *E. coli* peritonitis model

*E. coli* O18ac:K1:H7 [American Type Culture Collection (ATCC) 700973] bacterial culture was obtained from ATCC and reconstituted in LB growth medium. For peritonitis model, bacteria were subcultured in LB growth medium and collected in the logarithmic growth phase. Bacteria were pelleted and washed three times with sterile saline, and CFUs were estimated on the basis of optical density at 600 nm. Culture was serially diluted in sterile saline to a concentration of approximately 10^8^ CFUs/ml. An aliquot of inoculum was plated on LB agar plates to determine the final concentration. After pretreatment with vehicle or firsocostat for 1 hour, male C57BL/6J mice were inoculated with 100 μl of solution (approximately 10^7^ CFUs) or saline control and monitored for survival and humane end points. When mice reached humane end-point criteria or at the end of the experiment, peritoneal cavities were lavaged with 5 ml of sterile saline, which was serially diluted and plated on LB agar plates to estimate final CFU counts from *E. coli*–inoculated animals.

### BMDM culture

BMDMs were cultured as described previously (*7*). Briefly, bone marrow was isolated from the hindlimbs of mice and incubated with 0.83% (w/v) ammonium chloride for 5 min to lyse erythroid progenitors. Bone marrow cells were cultured in RPMI 1640 medium (Gibco) supplemented with 10% heat-inactivated fetal bovine serum (FBS) (Atlanta Biologicals), 2% Hepes (Gibco), 2% antibiotic-antimycotic (Gibco), and 10% L929-conditioned medium (L929 cells obtained from ATCC). Cells were cultured for 7 days, with medium changes every 3 to 4 days, before cells were switched to a medium lacking L929-conditioned medium. Cells were detached from petri dishes by brief incubation with 0.25% trypsin-EDTA (Gibco) and plated for analysis.

### hMDM culture

hMDMs were cultured from human peripheral blood mononuclear cells (PBMCs) enriched from buffy coats obtained from the Program for Non-Transfusable Material for In-Vitro Use of the American Red Cross (Columbus, OH). Buffy coat diluted 1:1 with sterile phosphate-buffered saline (PBS) was cleared of erythrocytes by differential centrifugation over Ficoll Paque Plus (GE Healthcare) using SepMate tubes (STEMCELL Technologies). Enriched PBMCs were pelleted by centrifugation at 300*g*, washed twice in sterile PBS, and resuspended for counting. Cells were resuspended in monocyte attachment medium (PromoCell) following the manufacturer’s instructions and allowed to adhere for 90 min. After two washes with monocyte attachment medium, adherent cells were cultured in RPMI 1640 (Gibco) supplemented with 2% Hepes (Gibco), 2% antibiotic-antimycotic (Gibco), 10% heat-inactivated FBS (Atlanta Biologicals), and recombinant human macrophage colony-stimulating factor (20 ng/ml; PeproTech) for 6 days. After 6 days, adherent monocyte-derived macrophages were detached using trypsin, counted, and plated for analysis.

### Peritoneal lavage and cell isolation

Cells were isolated from the peritoneal cavities of mice 6 or 24 hours after LPS inoculation. After confirmation of euthanasia, 5 ml of ice-cold sterile PBS with 5 mM EDTA was instilled in the peritoneal cavity, and the peritoneum was gently massaged to dislodge resident and infiltrating cells. Lavage fluid was recovered, and peritoneal cells were pelleted by centrifugation at 400*g* for 5 min. Supernatant lavage fluid was stored at −80°C, and cells were incubated in ACK lysis buffer to lyse erythrocytes. Cells were collected by centrifugation and resuspended in TRIzol (Invitrogen) before analysis.

### RNA sequencing

BMDMs (1 × 10^7^ cells) were plated on 10-cm petri dishes and allowed to adhere overnight. After 6-hour treatment with control or LPS (100 ng/ml), cells were washed once in ice-cold PBS, collected by centrifugation at 500*g* for 5 min, and lysed in RLT buffer (QIAGEN) before extraction of total RNA with the RNeasy Mini Kit (QIAGEN) and elution into nuclease-free water (Invitrogen). RNA concentration and purity were assessed on a NanoDrop 2000 spectrophotometer (Thermo Fisher Scientific). Library preparation and sequencing were performed by GENEWIZ (South Plainfield, NJ). Sample integrity was assessed on Agilent TapeStation before library preparation. One hundred fifty–base pair paired-end sequencing was performed using the Illumina HiSeq platform following the manufacturer’s instructions. Sequencing data were converted to Fastq files and demultiplexed. FastQC was used to check for read quality, trimmed reads were aligned to the mouse reference genome using STAR (*65*), and FeatureCounts (*66*) was used to tabulate reads across each gene. Gene expression analysis was performed using the edgeR workflow (*67*). Pathway, ontology, and related analyses of differentially expressed gene lists were performed using Enrichr (*33*, *34*).

### RNA isolation and quantitative reverse transcription PCR

RNA was isolated from approximately 4 × 10^5^ BMDMs lysed in RLT lysis buffer or from tissues homogenized in TRIzol using the RNeasy Mini Kit (QIAGEN) according to the manufacturer’s instructions. RNA was quantified using a NanoDrop spectrophotometer (Thermo Fisher Scientific), and complementary DNA (cDNA) libraries were generated using the iScript cDNA Synthesis Kit (Bio-Rad). Quantitative reverse transcription PCR was performed using SensiMix SYBR Green reagent (Bioline) and primer pairs for genes of interest on a CFX Connect real-time PCR instrument (Bio-Rad). Relative gene expression was determined using *B2m* or *Hprt* as a housekeeping gene. Primer sequences were verified against genes of interest using the National Center for Biotechnology Information (NCBI) Primer-BLAST.

### Immunoblot analysis

Approximately 2 × 10^6^ BMDMs were lysed on ice into radioimmunoprecipitation assay (RIPA) buffer containing cOmplete Mini Protease Inhibitor (Roche), phosphatase inhibitor cocktails (Sigma-Aldrich), 5 μM trichostatin A, and 10 mM nicotinamide (deacetylase inhibitors) and cleared by centrifugation at 20,000*g*. The protein content in cleared supernatants was estimated by bicinchoninic acid (BCA) assay (Pierce), and 10 to 50 μg of protein were denatured in Laemmli buffer, separated on a 6 to 15% gel by SDS–polyacrylamide gel electrophoresis, and transferred to nitrocellulose membranes. Membranes were blocked in 5% bovine serum albumin in tris-buffered saline (TBS) and stained overnight at 4°C with primary antibodies. Membranes were washed in TBS with 0.1% Tween 20 and stained with IRDye-conjugated secondary antibodies (1:10,000; LI-COR Biosciences), and protein was visualized on an Odyssey Imager (LI-COR Biosciences).

For histone immunoblotting, approximately 4 × 10^6^ to 5 × 10^6^ BMDMs were lysed in extraction buffer [PBS with 0.5% (v/v) Triton X-100, 2 mM phenylmethylsulfonyl fluoride (PMSF), and 0.02% (w/v) NaN_3_ supplemented with protease, phosphatase, and deacetylase inhibitors as above] on ice for 10 min, and nuclei were pelleted by centrifugation at 6500*g* for 10 min. Nuclei were resuspended in 100 μl of acid extraction buffer (0.2 M HCl in H_2_O with protease, phosphatase, and deacetylase inhibitors), and histones were extracted at 4°C overnight. Samples were neutralized by addition of 2 M NaOH in H_2_O and cleared by centrifugation at 6500*g* for 10 min. The protein content of supernatants (containing extracted histones) was estimated by BCA assay, and 2 to 10 μg of samples were assayed by immunoblot.

Avidin pulldown of biotin-containing proteins was done as previously described (*25*). Briefly, BMDMs were lysed in RIPA buffer containing cOmplete Mini Protease Inhibitor (Roche), phosphatase inhibitor cocktails (Sigma-Aldrich), 5 μM trichostatin A, and 10 mM nicotinamide (deacetylase inhibitors) and cleared by centrifugation at 20,000*g*. Protein content in cleared supernatants was estimated by BCA assay (Pierce), and approximately 300 μg of total protein was incubated with streptavidin-conjugated agarose (Strep-Tactin Superflow Plus, QIAGEN) overnight to enrich biotin-containing proteins. Beads were pelleted by centrifugation at 2000*g*, washed two times in RIPA buffer, and heated to 60°C for 15 min in Laemmli buffer, and samples were assayed by immunoblot.

For preparation of nuclear and cytoplasmic extracts, the protocol of Schreiber *et al.* (*68*) was followed with minor modifications. Briefly, approximately 1 × 10^7^ BMDMs were plated on 10-cm petri dishes and allowed to adhere overnight. After treatments as indicated, cells were scraped into sterile, ice-cold PBS and pelleted by centrifugation for 5 min at 400*g*. PBS was removed, and the cell pellet was resuspended in cytoplasmic lysis buffer [10 mM Hepes (pH 7.9), 10 mM KCl, 0.1 mM EDTA, 0.1 mM EGTA, 1 mM dithiothreitol (DTT), and 0.5 mM PMSF supplemented with protease, phosphatase, and deacetylase inhibitors as described above]. Samples were allowed to swell on ice for 15 min before the addition of NP-40 (10% in water) at ^1^/_16_ the total volume and disruption by vortexing for 10 s. Samples were centrifuged at 6600*g* for 5 min, and the supernatant was retained as the cytoplasmic fraction. Pelleted nuclei were resuspended in nuclear buffer [20 mM Hepes (pH 7.9), 400 mM NaCl, 1 mM EDTA, 1 mM EGTA, 1 mM DTT, and 1 mM PMSF supplemented with protease, phosphatase, and deacetylase inhibitors as described above] at one-fourth the volume of cytoplasmic buffer and mixed at 1100 rpm in a tube shaker at 4°C for 15 min. The insoluble material was pelleted by centrifugation at 6600*g* for 10 min, and supernatants were retained as nuclear extract. Cytoplasmic and nuclear extracts were frozen at −80°C before use, and the protein content was estimated using the method of Bradford (*69*). Samples (10 to 25 μg) were assayed by immunoblot.

For cell surface biotinylation enrichment of surface-associated proteins, approximately 10^7^ BMDMs were seeded on 10-cm petri dishes and allowed to adhere overnight. After stimulation with control or LPS for 6 hours, cells were washed with ice-cold PBS before incubation with a solution of Pierce EZ-Link Sulfo-NHS-LC-Biotin (Thermo Fisher Scientific) in PBS at 4°C for 1 hour with rocking. Biotin was quenched with the addition of 100 mM glycine in PBS before cells were washed in PBS and lysed into RIPA buffer with protease and phosphatase inhibitors. DNA was sheared by brief sonication before protein concentration was estimated using BCA assay. An aliquot of each sample was lysed in Laemmli buffer as an input control before 400 μg of each sample was incubated with streptavidin-conjugated agarose (Strep-Tactin Superflow Plus, QIAGEN) overnight. Samples were pelleted by gentle centrifugation, washed twice with fresh RIPA buffer, and eluted into 2× Laemmli buffer at 60°C for 15 min before analysis by immunoblot.

### Antibodies

Antibodies used were the following: anti-ACC (1:500; C83B10, Cell Signaling Technology, 3676), anti–β-actin (1:1000; LI-COR Biosciences, 926-42210), anti-NFκB p65 (1:500; Cell Signaling Technology, 8242), anti–phospho-NFκB p65 (S536) (1:500; Cell Signaling Technology, 3033), anti-ERK p42/p44 (1:1000; Cell Signaling Technology, 4696), anti–phospho-ERK p42/p44 (T202/Y204) (1:1000; Cell Signaling Technology, 4377), anti–glyceraldehyde-3-phosphate dehydrogenase (GAPDH) (1:1000; Cell Signaling Technology, 5174), anti–phospho-STAT6 (Y641) (1:1000; Cell Signaling Technology, 9361), anti-STAT6 (1:1000; Cell Signaling Technology, 9362), anti-vinculin (1:1000; Cell Signaling Technology, 13901), anti-FASN (1:1000; Cell Signaling Technology, 3180), anti-arginase (1:500; Santa Cruz Biotechnology, sc-18351), anti–acetyl-H3 (K27) (1:500; Active Motif, 39133), anti-H3 (1:5000; Abcam, ab1791), rodent OXPHOS antibody cocktail (1:250; Abcam, ab110413), and anti-GLUT1 (1:1000; Novus Biologicals, NB110-39113).

### Extracellular flux analysis

Extracellular flux analysis was performed as previously described (*7*). Briefly, BMDMs were seeded into a 24-well or 96-well XF culture plate (Agilent Technologies) and allowed to adhere overnight. To assess glycolytic capacity, cells were subjected to a glycolytic stress test, which measures the ECAR as established previously. At the end of the experimental treatment, cells were switched to unbuffered, glucose-free Dulbecco’s modified Eagle’s medium (DMEM) (Sigma-Aldrich, D5030) supplemented with 143 mM NaCl (Sigma-Aldrich) and 2 mM l-glutamine (Gibco) (pH 7.35) at 37°C. After basal ECAR measurements, glucose (20 mM), oligomycin (1 μM), and 2-deoxyglucose (80 mM) were sequentially injected every four measurements and ECAR was recorded. Basal glycolysis was measured by subtracting the average of the post–2-deoxyglucose measurements from the average of the post-glucose measurements. Glycolytic capacity was calculated by subtracting the average of the post–2-deoxyglucose measurements from the average of the post-oligomycin measurements. For modified glycolytic stress test, glucose was co-injected with or without LPS (100 ng/ml) and ECAR followed for 6 hours before injection of oligomycin and 2-deoxyglucose.

To assess mitochondrial function, cells were subjected to a mitochondrial stress test as previously described (*7*). Briefly, at the end of the experiment, the medium was changed to DMEM with pyruvate (pH 7.35 at 37°C; 12800017, Thermo Fisher Scientific) and cells were equilibrated for 30 min. After basal OCR measurements, compounds to modulate cellular respiratory function [1 μM oligomycin (to inhibit ATP synthase), 2 μM BAM15 (to cause mitochondrial uncoupling), 1 μM antimycin A, and 100 nM rotenone (to inhibit mitochondrial respiration)] were injected into the plate, and OCR was recorded for three to four measurements between injections. Basal respiration was calculated by subtracting the average of the post–antimycin A and rotenone measurements from the average of the first three measurements. The maximum respiratory capacity was calculated by subtracting the average of the post–antimycin A and rotenone measurements from the average of the post-BAM15 measurements. The reserve capacity was calculated by subtracting the average of the basal measurements from the average of the post-BAM15 measurements.

### Cytokine measurements

Cytokine concentrations were quantified by enzyme-linked immunosorbent assay (ELISA). For in vitro experiments, 4 × 10^5^ BMDMs were seeded in 24-well plates and allowed to adhere overnight before experimental treatment. At the end of the experiment, the supernatant medium was collected and stored at −80°C for further analysis. For in vivo experiments, the peritoneal lavage supernatant or plasma was collected and stored at −80°C for further analysis. Cytokine concentrations were determined using commercial ELISA kits (Invitrogen) against IL-6 and IL-1β according to the manufacturer’s instructions, and absorbance was recorded on a Synergy HTX plate reader (BioTek Instruments).

### Cell-based glucose uptake assay

BMDMs were plated at 5 × 10^4^ per well in a black, clear-bottom 96-well plate and allowed to adhere overnight. After stimulation as indicated, the uptake of the fluorescent glucose analog 2-NDBG in glucose-free RPMI 1640 (11879020, Gibco) was assessed by fluorescence according to the manufacturer’s instructions (Glucose Uptake Cell-Based Assay Kit, Cayman Chemical).

### H_2_DFCDA ROS assay

BMDMs were plated at 5 × 10^4^ per well in a black, clear-bottom 96-well plate and allowed to adhere overnight. After LPS stimulation, cells were incubated with 2',7'-dichlorodihydrofluorescein diacetate (H_2_DFCDA) for 30 min before fluorescence measurement according to the manufacturer’s instructions (ROS Assay Kit, BioVision). Fluorescence values were corrected for background (unstained cells) and represented as percent of unstimulated flox control.

### Lactate assay

Lactate concentration was determined from supernatants of cells unstimulated or after 6 hours of stimulation with LPS or LTA. Lactate concentration in serum-free supernatants was determined using the BioVision Colorimetric/Fluorometric Lactate Assay Kit (K607) according to the manufacturer’s instructions.

### SDH activity assay

BMDMs (~1 × 10^7^) were stimulated with vehicle or LPS for 6 hours; cells were scraped into SDH assay buffer supplemented with protease, phosphatase, and deacetylase inhibitors; and samples were cleared by centrifugation at 20,000*g* for 15 min. SDH activity of cleared supernatants was determined according to the manufacturer’s instructions (SDH Activity Assay Kit, Sigma-Aldrich) and normalized to protein concentration as determined by BCA assay.

### NADPH assay

BMDMs (2 × 10^6^) were plated in six-well plates and allowed to adhere overnight. After stimulation with LPS, cells were washed with PBS and lysed into 150 μl of PBS and 150 μl of bicarbonate lysis buffer (100 mM sodium carbonate, 20 mM sodium bicarbonate, 10 mM nicotinamide, and 0.05% Triton X-100). Samples were digested in acidic or basic solution to allow for measurement of NADP^+^ or NADPH. After neutralization, levels of oxidized or reduced NADPH were measured with the NADP/NADPH-Glo Assay (Promega) and luminescence was measured on a Synergy HTX plate reader (BioTek Instruments).

### Bacterial killing assay

BMDMs (2 × 10^5^) were seeded in 24-well plates and allowed to adhere overnight. *E. coli* was grown overnight in LB, and OD_600_ (optical density at 600 nm) was used to determine bacterial counts. On the day of the experiment, *E. coli* was washed in RPMI 1640 supplemented with 10% FBS and plated on BMDMs at a multiplicity of infection of 1. Plates were spun at 500*g* for 5 min to facilitate bacterial engulfment. Plates were placed in incubator (37°C, 5% CO_2_) for 30 min. After 30 min, BMDMs were washed three times in RPMI 1640 supplemented with gentamicin (50 μg/ml; Gibco) to remove non-engulfed bacteria. One plate of cells was harvested at 30-min time point and lysed in sterile distilled H_2_O for 30 min before being serially diluted and plated on LB agar plates for T0 CFUs. The second, T2 plate, was placed back into the incubator for 2 hours in RPMI 1640 supplemented with 10% FBS and gentamicin (50 μg/ml). After 2 hours, T2 plates were lysed and plated in the same method as T0 plates. Percent killing was calculated from CFUs using the formula [(T0 − T2)/T0]*100.

### LC-MS lipidomics

BMDMs were plated on 10-cm petri dishes and allowed to adhere overnight. Cells were treated with control medium or medium containing LPS (100 ng/ml) for 6 hours before cells were washed, scraped into PBS, and counted. Approximately 2.5 × 10^6^ cells were lysed in high-performance LC (HPLC)–grade water (Sigma-Aldrich) and spiked with deuterated lipid standards (SPLASH LIPIDOMIX, Avanti Polar Lipids) before modified Bligh-Dyer extraction. Lipids were extracted in a 1:1:1 ratio of water/methanol/chloroform in glass culture tubes, which was homogenized by vortexing, before centrifugation at 800*g* for 10 min to accelerate phase separation. The organic phase was removed, and extraction was performed twice more for a total of three extractions. Combined organic phases were dried under nitrogen gas and resuspended in 100 μl of HPLC-grade methanol before LC-MS analysis.

LC-MS analysis was performed on a Q Exactive Orbitrap high-resolution mass spectrometer (Thermo Fisher Scientific) coupled to a Vanquish UHPLC system essentially as previously described (*70*). Samples (10 μl) were separated by reverse phase on a 100 mm × 4.6 mm C18 column (5 μm, 120 Å; Thermo Fisher Scientific, Acclaim 120) using a two-phase solvent system consisting of mobile phase A (50% acetonitrile, 50% water, and 0.1% formic acid with 10 mM ammonium formate) and mobile phase B (88% isopropanol, 10% acetonitrile, 2% water, and 0.02% formic acid with 2 mM ammonium formate) at 400 μl/min using the following gradient: 0 to 4 min, 30 to 60% B; 4 to 10 min, 60 to 80% B; 10 to 15 min, 80 to 90% B; 15 to 24 min, 90 to 100% B; 24 to 27 min, 100% B; 27 to 27.1 min, 100 to 30% B; and 27.1 to 31 min, 30% B. Mass spectra were collected in positive ionization mode using a Top5 Full MS data-dependent MS/MS (ddMS^2^) method with the following settings: full MS settings: resolution of 35,000, automatic gain control (AGC) target of 1 × 10^5^, maximum injection time (IT) of 128 ms, and scan range of 200 to 1500 mass/charge ratio (*m/z*); ddMS^2^ settings: resolution of 17,500, AGC target of 2 × 10^5^, maximum IT of 64 ms, loop count of 5, and normalized collision energy of 40. Data analysis and assignment of lipid species were performed using LipidSearch 4.1.16 (Thermo Fisher Scientific) with the following settings: search—database: Q Exactive, precursor tolerance of 5.0 parts per million (ppm), and product tolerance of 8.0 ppm; alignment—alignment method, mean and retention time tolerance, 0.25 min.

### Cholesterol measurements

Cholesterol was measured using LC-MS on a Q Exactive Orbitrap high-resolution mass spectrometer (Thermo Fisher Scientific) coupled to a Vanquish UHPLC system. Samples spiked with SPLASH LIPIDOMIX (containing cholesterol-*d*7) extracted as described for lipidomics were analyzed by reversed-phase separation on a 100 mm × 4.6 mm, 5 μm, C18 column (Kinetex, Phenomenex) consisting of mobile phase A (85% methanol, 15% water, and 5 mM ammonium acetate) and mobile phase B (100% methanol and 5 mM ammonium acetate) at a flow rate of 200 μl/min using the following gradient as previously described (*71*): 2 min, 50% B; 2 to 5 min, 50 to 100% B; 5 to 18 min, 100% B; 18 to 22 min, 100 to 0% B; 22 to 27 min, 0% B; 27 to 28 min, 0 to 50% B; and 28 to 35 min, 50% B. Mass spectra were collected in positive ionization mode using parallel reaction monitoring with an inclusion list for cholesterol and the deuterated cholesterol-*d*7 standard. Data were analyzed using the Xcalibur Quan Browser, abundance was corrected for extraction efficiency using recovery of cholesterol-*d*7, and data were presented relative to flox control condition.

### ^14^C-acetate incorporation

BMDMs (approximately 2 × 10^6^ per plate) were seeded on petri dishes and allowed to adhere overnight. Serum-free RPMI 1640 was spiked with [1,2-^14^C]sodium acetate (PerkinElmer, NEC553050UC) to a final concentration of 1 μCi/ml with or without LPS (100 ng/ml) and incubated at 37°C for 6 hours. At the end of the experiment, medium was aspirated and cells were washed twice with ice-cold PBS and scraped into lysis buffer (PBS + 0.1% Triton X-100 with protease inhibitors and 0.5 mM DTT). Lysates (200 μl) were extracted with 0.5 ml of acidified methanol, 0.25 ml of chloroform, and 0.25 ml of 200 mM NaCl, and the remaining sample was saved for estimation of protein concentration. After vortexing, phase separation was accelerated by centrifugation at 1000*g* for 2 min. Incorporation of ^14^C-labeled acetate into the lipid-soluble fraction was determined by scintillation counting and normalized to protein content of samples.

### Statistical analysis

Statistical analysis was performed using Prism 9 (GraphPad Software). Comparisons between two groups were conducted by Welch’s or Student’s *t* test or Mann-Whitney test, while comparisons of more than two groups were made by one-way analysis of variance (ANOVA) with post hoc testing of differences between individual groups, where appropriate. Statistical details are provided in individual figure legends. Outliers were assessed using the ROUT outlier test in Prism 9 (*Q* = 5%) and excluded from analysis. Data are represented as means ± SEM.

### Study approval

All animal studies described were approved by the Animal Care and Use Committee of the University of Virginia, under protocol number 3444. The use of anonymized human buffy coat for hMDM isolation was approved by agreement with the Institutional Review Board of the American Red Cross (Columbus, OH).

## Supplementary Materials

### This PDF file includes:

Figs. S1 to S6 Table S1 Data S2. Uncropped_Gels_Blots: Summary PDF of uncropped gels and blots.

## Notes

View/request a protocol for this paper from *Bio-protocol*.
