# Supplementary material for: Macrophage acetyl-CoA carboxylase regulates acute inflammation through control of glucose and lipid metabolism
Source: Sci Adv. 2022 Nov 23;8(47):eabq1984. doi: 10.1126/sciadv.abq1984 (PMC9683712; doi:10.1126/sciadv.abq1984)

Science Advances
AAAS

Supplementary Materials for

Macrophage acetyl-CoA carboxylase regulates acute inflammation through control of glucose and lipid metabolism

Scott Yeudall *et al.*

Corresponding author: Norbert Leitinger, nl2q@virginia.edu

Sci. Adv. **8**, eabq1984 (2022)
DOI: 10.1126/sciadv.abq1984

The PDF file includes:

Figs. S1 to S6
Table S1
Legends for data S1 and S3
Data S2. Uncropped_Gels_Blots: Summary PDF of uncropped gels and blots.

Other Supplementary Material for this manuscript includes the following:

Data S1 and S3

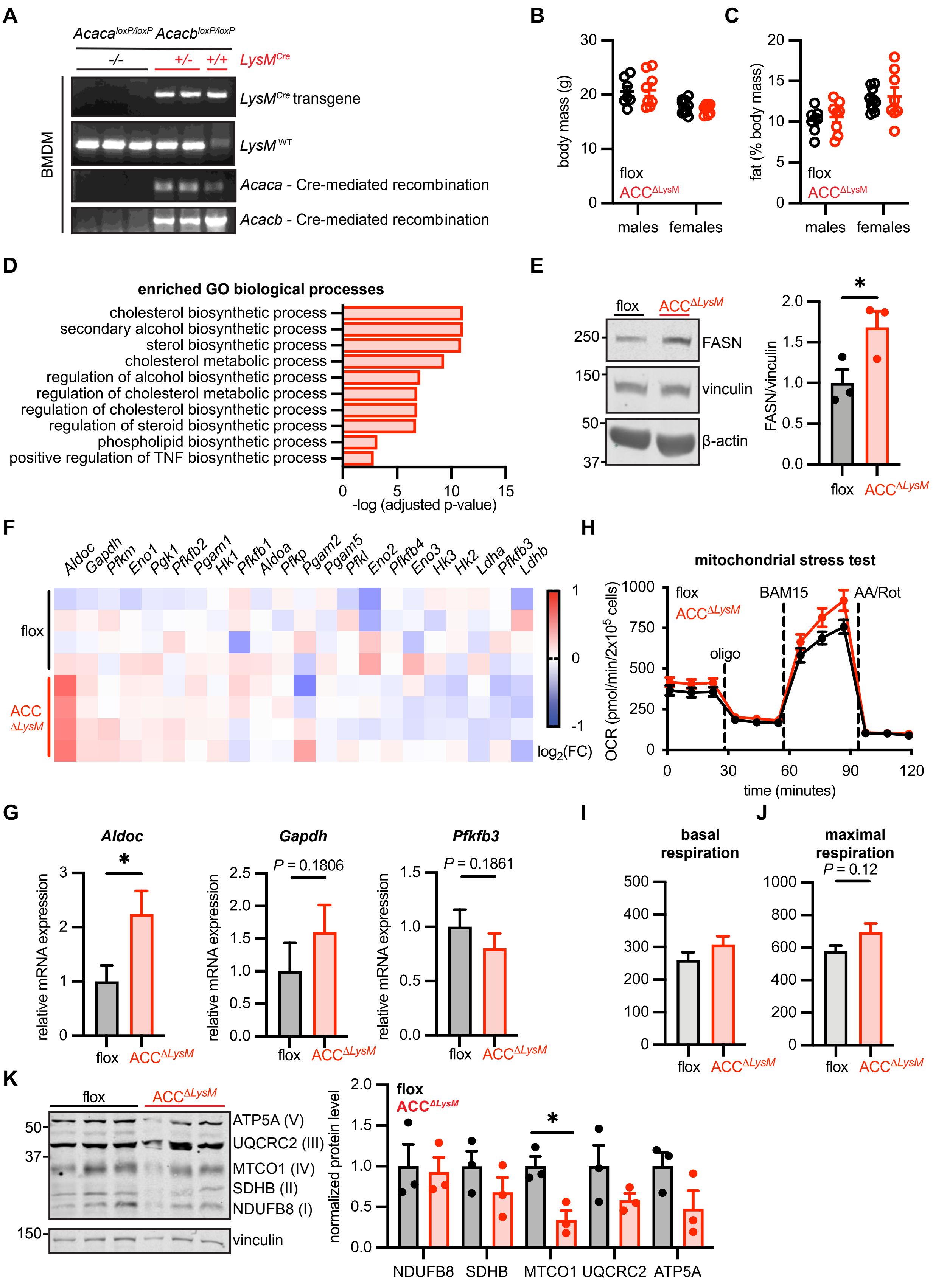

A
Acaca loxP/loxP Acacb loxP/loxP
-/-
+/-
+/+
LysMCre
BMDM
LysMCre transgene
LysM WT
Acaca - Cre-mediated recombination
Acacb - Cre-mediated recombination
B
body mass (g)
flox
ACCΔLysM
males
females
C
fat (% body mass)
D
enriched GO biological processes
cholesterol biosynthetic process
secondary alcohol biosynthetic process
sterol biosynthetic process
cholesterol metabolic process
regulation of alcohol biosynthetic process
regulation of cholesterol metabolic process
regulation of cholesterol biosynthetic process
regulation of steroid biosynthetic process
phospholipid biosynthetic process
positive regulation of TNF biosynthetic process
-log (adjusted p-value)
E
FASN
vinculin
β-actin
FASN/vinculin
F
Aldoc Gapdh Pfkm Eno1 Pgk1 Pfkfb2 Pgam1 Hk1 Pfkfb1 Aldoa Pfkp Pgam2 Pgam5 Pfkl Eno2 Pfkfb4 Eno3 Hk3 Hk2 Ldha Pfkfb3 Ldhb
log2(FC)
H
mitochondrial stress test
OCR (pmol/min/2x105 cells)
oligo
BAM15
AA/Rot
time (minutes)
G
Aldoc
Gapdh
Pfkfb3
relative mRNA expression
P = 0.1806
P = 0.1861
I
basal respiration
J
maximal respiration
P = 0.12
K
ATP5A (V)
UQCRC2 (III)
MTCO1 (IV)
SDHB (II)
NDUFB8 (I)
vinculin
normalized protein level
NDUFB8
SDHB
MTCO1
UQCRC2
ATP5A

Supplementary Figure 1, related to Figure 1.
A. Representative genotyping of BMDM samples from flox or ACC$^{\Delta LysM}$ mice.
B. Body mass of flox and ACC$^{\Delta LysM}$ mice at 8 weeks of age (male flox, n = 7; male ACC$^{\Delta LysM}$, n = 8; female flox, n = 10; female ACC$^{\Delta LysM}$, n = 8).
C. Fat as percentage of total body mass in flox and ACC$^{\Delta LysM}$ mice at 8 weeks of age (male flox, n = 7; male ACC$^{\Delta LysM}$, n = 8; female flox, n = 10; female ACC$^{\Delta LysM}$, n = 8).
D. Enriched GO Biological Processes from unstimulated ACC$^{\Delta LysM}$ BMDMs.
E. Representative immunoblot analysis and quantification of fatty acid synthase (FASN) in unstimulated BMDMs from flox or ACC$^{\Delta LysM}$ mice (n = 3 mice per group). Vinculin and β-actin were used as loading controls.
F. Heatmap showing fold expression of glycolytic enzyme genes in unstimulated flox and ACC$^{\Delta LysM}$ BMDMs (n = 4).
G. Expression of glycolytic genes *Aldoc, Gapdh,* and *Pfkfb3* in unstimulated flox or ACC$^{\Delta LysM}$ BMDMs (n = 4).
H. Mitochondrial stress test (MST) of BMDMs from flox or ACC$^{\Delta LysM}$ mice (n = 4).
I. Basal respiration (calculated as average basal OCR – average post-AA/rot OCR) of flox or ACC$^{\Delta LysM}$ BMDMs (n = 4).
J. Maximal respiration (calculated as average post-BAM15 OCR – average post-AA/rot OCR) of flox or ACC$^{\Delta LysM}$ BMDMs (n = 4).
K. Immunoblot analysis of mitochondrial oxidative phosphorylation complexes (n = 3 mice per group). Vinculin was used as loading control.
Data are represented as mean±SEM. Significance determined by one-tailed Welch's t-test (B, C, E, G, I, J, K). * $P < 0.05$.

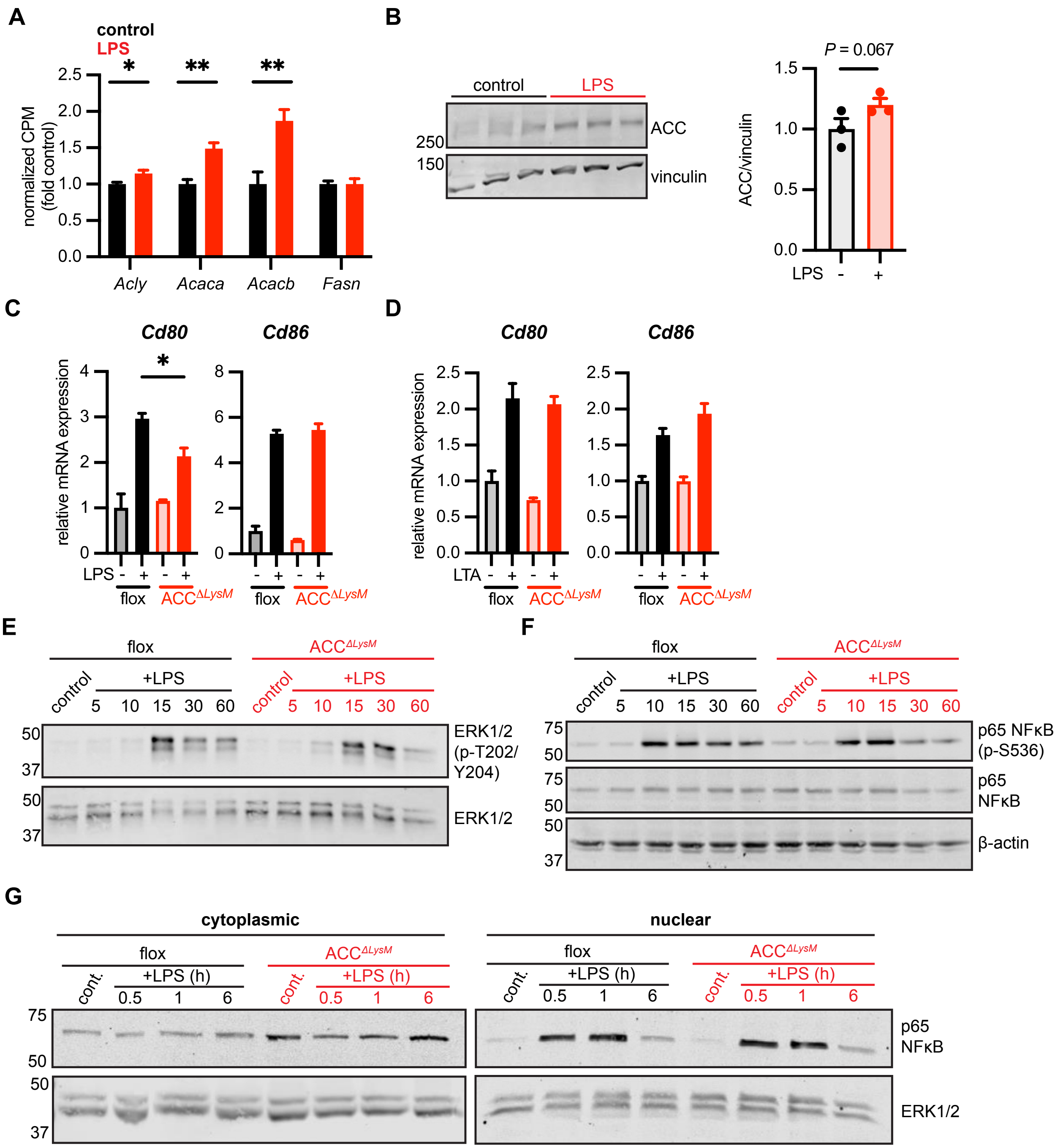

Supplementary Figure 2, related to Figure 2.

A. Normalized gene expression (normalized counts per million) of *Acly, Acaca, Acacb,* and *Fasn* of BMDMs treated with control or LPS (100ng/mL) for 6 hours (n = 4).

B. Immunoblot and semi-quantification of ACC in BMDMs treated with LPS (100ng/mL) for 6 hours (n = 3). Vinculin was used as a loading control.

C. Relative mRNA expression of *Cd80* and *Cd86* in flox or ACC$^{\Delta LysM}$ BMDMs treated with LPS (100ng/mL) for 6 hours (n = 4).

D. Relative mRNA expression of *Cd80* and *Cd86* in flox or $ACC^{\Delta LysM}$ BMDMs treated with LTA (1μg/ml) for 6 hours (n = 4).
E. Immunoblot of ERK1/2 phosphorylation at T202/Y204 in flox or $ACC^{\Delta LysM}$ BMDMs unstimulated or after LPS stimulation (100ng/ml) for 5-60 minutes. Representative of two independent experiments.
F. Immunoblot of p65 NF-κB phosphorylation at S536 in flox or $ACC^{\Delta LysM}$ BMDMs unstimulated or after LPS stimulation (100ng/ml) for 5-60 minutes. Representative of two independent experiments. β-actin was used as a loading control.
G. Immunoblot of total p65 NF-κB in cytoplasmic or nuclear extracts from flox or $ACC^{\Delta LysM}$ BMDMs unstimulated or after LPS stimulation (100ng/ml) for 0.5, 1, or 6 hours. Representative of two independent experiments. ERK1/2 was used as a loading control.
Data are represented as mean ± SEM. Significance determined by one-tailed Welch's t-test (A, B) or One-Way ANOVA (C,D) . * $P < 0.05$, ** $P < 0.01$.

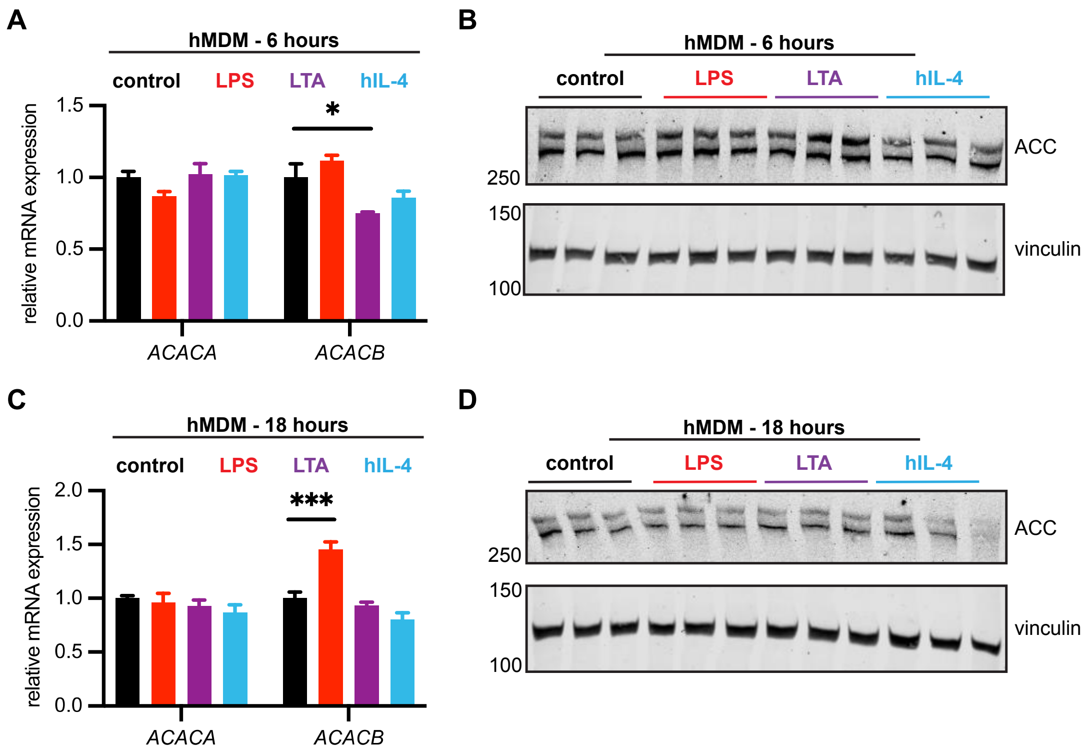

Supplementary Figure 3, related to Figures 2 and 4.

A. Relative mRNA expression of *ACACA* and *ACACB* in human MDMs treated with LPS (100ng/mL), LTA (1µg/ml) or hIL-4 (10ng/mL) for 6 hours (n = 4).

B. Immunoblot of ACC in human MDMs treated with LPS (100ng/mL), LTA (1µg/ml) or hIL-4 (10ng/mL) for 6 hours (n = 3). Vinculin was used as a loading control.

C. Relative mRNA expression of *ACACA* and *ACACB* in human MDMs treated with LPS (100ng/mL), LTA (1µg/ml) or hIL-4 (10ng/mL) for 18 hours (n = 4).

D. Immunoblot of ACC in human MDMs treated with LPS (100ng/mL), LTA (1µg/ml) or hIL-4 (10ng/mL) for 18 hours (n = 3). Vinculin was used as a loading control.

Data are represented as mean±SEM. Significance determined by One-Way ANOVA (A, C). * $P < 0.05$, *** $P < 0.001$.

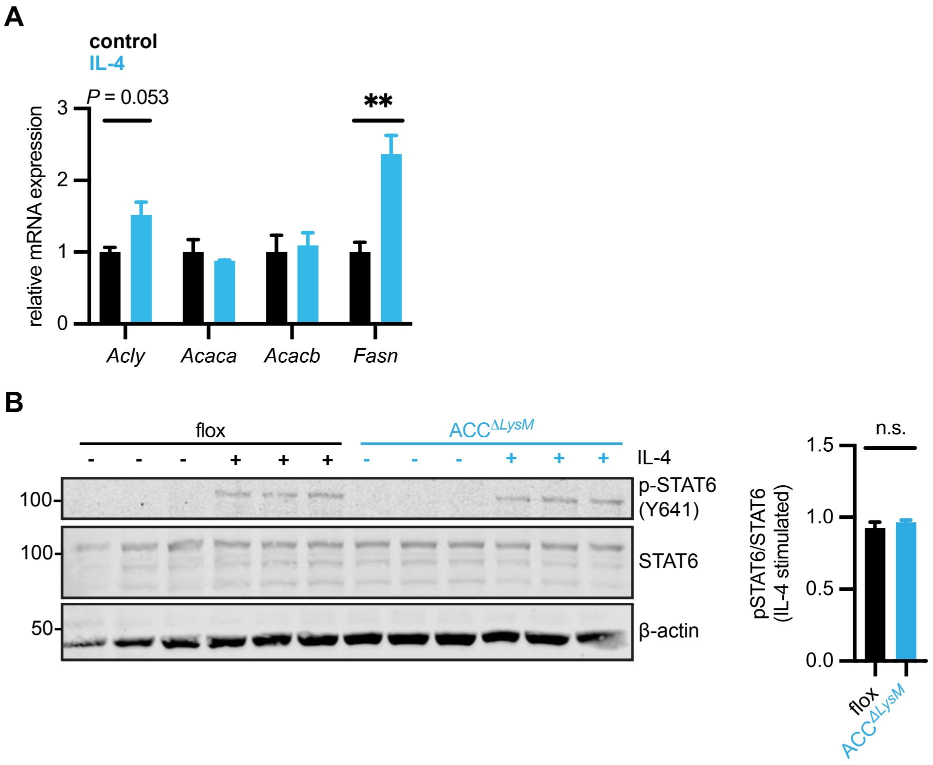

Supplementary Figure 4, related to Figure 4

A. Relative mRNA expression of *Acly, Acaca, Acacb,* and *Fasn* in BMDMs stimulated with IL-4 (10ng/mL) for 6 hours (n = 4).

B. Whole-cell immunoblot analysis of STAT6 phosphorylation at Y64 in flox or ACC$^{\Delta LysM}$ BMDMs stimulated with IL-4 (10ng/ml) for 6 hours (n = 3 mice per group). B-actin was used as a loading control.

Data are represented as mean±SEM. Significance determined by one-tailed Welch's t-test (A, B).

** $P < 0.01$

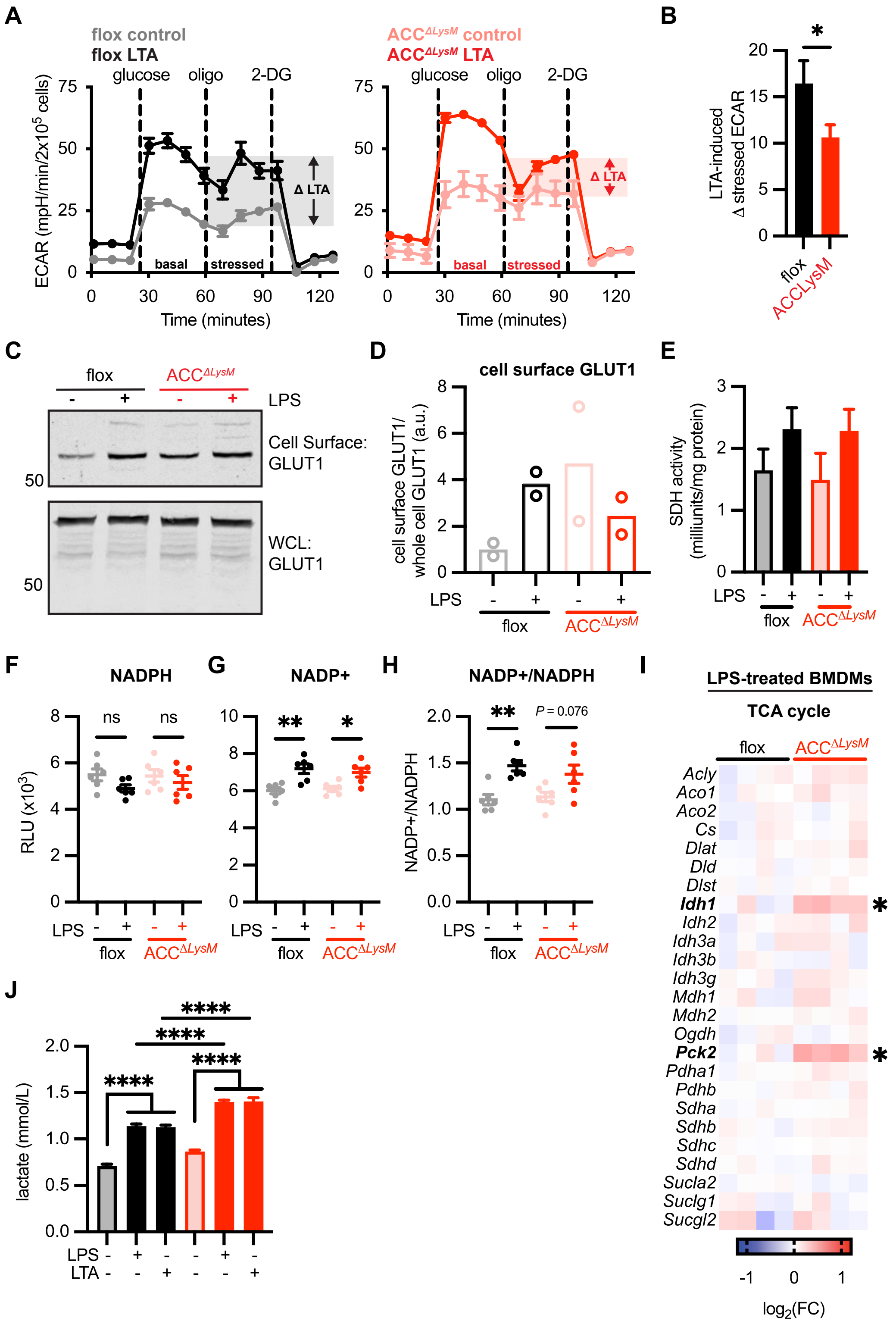

Supplementary Figure 5, Related to Figure 5

A. Glycolytic stress test of flox (black, left) or ACC$^{\Delta LysM}$ (red, right) BMDMs after stimulation with LTA for 16 hours. Shaded areas represent the LT+LTA-induced increase in stressed (post-oligomycin) ECAR (n = 5).

B. Quantification of LTA-induced change in stressed ECAR in flox or ACC$^{\Delta LysM}$ BMDMs after 16-hour stimulation with LPS (n = 5).
C. Representative immunoblot and **D.** semi-quantification of cell-surface and whole cell GLUT1 of flox or ACC$^{\Delta LysM}$ BMDMs treated with LPS for 6 hours subjected to cell-surface biotinylation protocol (n = 2 biological replicates).
D. Succinate dehydrogenase activity of flox or ACC$^{\Delta LysM}$ BMDMs treated with LPS for 6 hours (n = 6).
F. NADPH and **G.** NADP+ levels in BMDMs from flox or ACC$^{\Delta LysM}$ BMDMs treated with LPS for 6 hours (n = 6).
H. NADP+/NADPH ratio in BMDMs from flox or ACC$^{\Delta LysM}$ BMDMs treated with LPS for 6 hours (n = 6).
I. Heat map of relative expression of genes related to the tricarboxylic acid (TCA) cycle in LPS-treated flox and ACC$^{\Delta LysM}$ BMDMs (n = 4).
J. Supernatant lactate of flox or ACC$^{\Delta LysM}$ BMDMs unstimulated or after 6 hours of LPS or LTA stimulation (n = 6).
Data are represented as mean±SEM. Significance determined by One-Way ANOVA (C, E, G, H, J) or one-tailed Welch's t-test (B,I) or. * $P < 0.05$, ** $P < 0.01$, **** $P < 0.0001$.

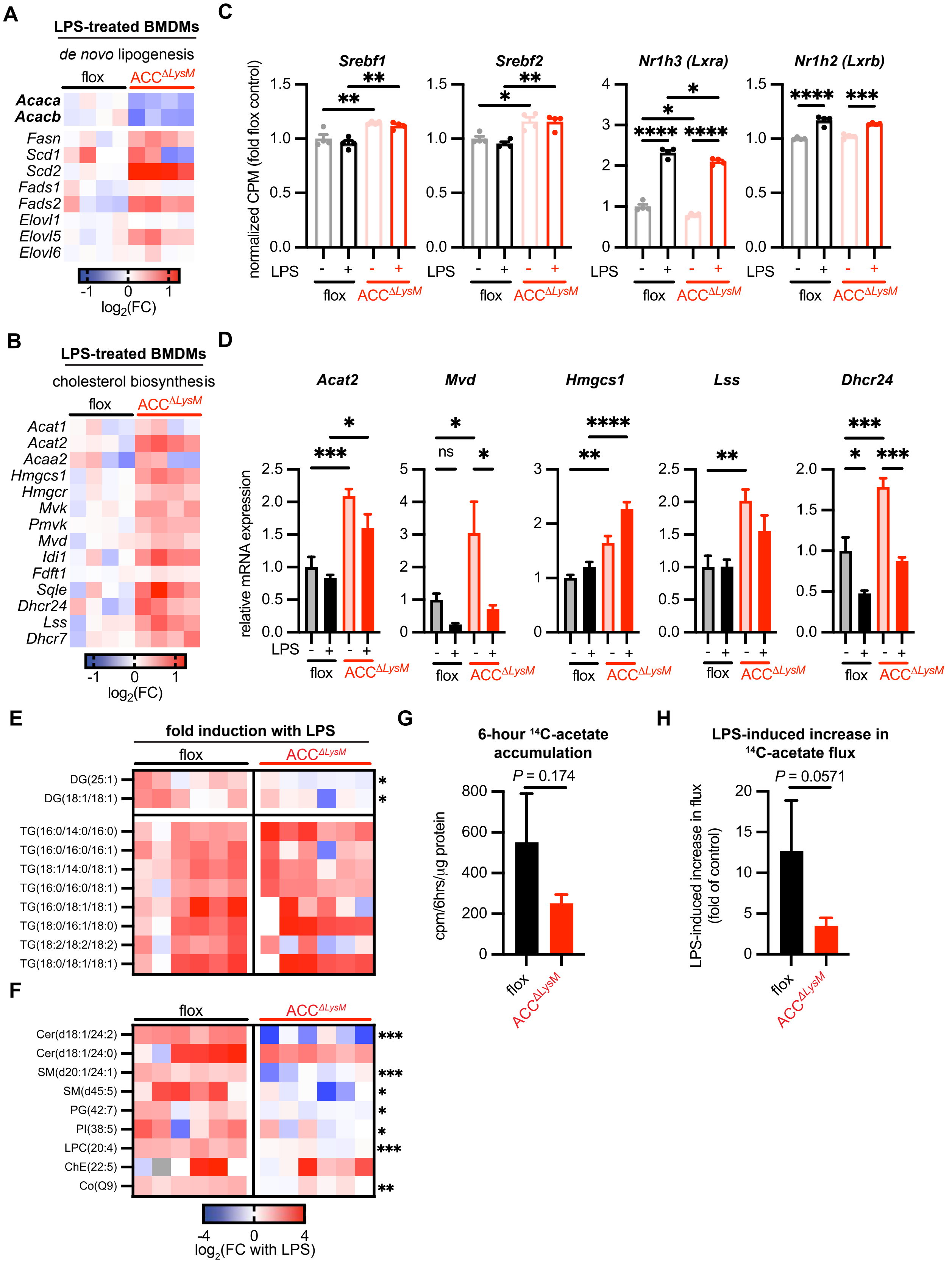

A
LPS-treated BMDMs
de novo lipogenesis
flox
ACCΔLysM
Acaca
Acacb
Fasn
Scd1
Scd2
Fads1
Fads2
Elovl1
Elovl5
Elovl6
-1
0
1
log2(FC)
C
Srebf1
Srebf2
Nr1h3 (Lxra)
Nr1h2 (Lxrb)
normalized CPM (fold flox control)
LPS
flox
B
LPS-treated BMDMs
cholesterol biosynthesis
Acat1
Acat2
Acaa2
Hmgcs1
Hmgcr
Mvk
Pmvk
Mvd
Idi1
Fdft1
Sqle
Dhcr24
Lss
Dhcr7
D
Acat2
Mvd
Hmgcs1
Lss
Dhcr24
relative mRNA expression
ns
E
fold induction with LPS
DG(25:1)
DG(18:1/18:1)
TG(16:0/14:0/16:0)
TG(16:0/16:0/16:1)
TG(18:1/14:0/18:1)
TG(16:0/16:0/18:1)
TG(16:0/18:1/18:1)
TG(18:0/16:1/18:0)
TG(18:2/18:2/18:2)
TG(18:0/18:1/18:1)
F
Cer(d18:1/24:2)
Cer(d18:1/24:0)
SM(d20:1/24:1)
SM(d45:5)
PG(42:7)
PI(38:5)
LPC(20:4)
ChE(22:5)
Co(Q9)
-4
0
4
log2(FC with LPS)
G
6-hour 14C-acetate accumulation
P = 0.174
cpm/6hrs/μg protein
H
LPS-induced increase in 14C-acetate flux
P = 0.0571
LPS-induced increase in flux (fold of control)

Supplementary Figure 6, related to Figure 6.
A. Heat map of relative expression of genes in the *de novo* lipogenesis pathway in LPS-treated flox and $ACC^{\Delta LysM}$ BMDMs (n = 4).
B. Heat map of relative expression of genes in the cholesterol biosynthesis pathway in LPS-treated flox and $ACC^{\Delta LysM}$ BMDMs (n = 4).
C. Relative expression of transcription factor genes *Srebf1*, *Srebf2*, *N21h3*, and *Nr1h2* in flox and $ACC^{\Delta LysM}$ BMDMs after control and LPS stimulation (n = 4).
D. Expression of cholesterol pathway genes from in flox and $ACC^{\Delta LysM}$ BMDMs after control and LPS stimulation was confirmed by qPCR (n = 4).
E. Heat map of significantly increased diacylglycerol (DAG) and triacylglycerol (TAG) species in LPS-stimulated flox and $ACC^{\Delta LysM}$ BMDMs, relative to respective unstimulated cells (n = 6).
F. Heat map of significantly increased ceramide, sphingomyelin, and other lipid species in LPS-stimulated flox and $ACC^{\Delta LysM}$ BMDMs, relative to respective unstimulated cells (n = 6).
G. Accumulation of ^{14}C-acetate signal in the lipid-soluble fraction of flox or $ACC^{\Delta LysM}$ BMDMs after 6 hours (n = 4).
H. BMDMs were stimulated with control or LPS (100ng/ml) for 4 hours, before corresponding media containing ^{14}C-acetate was added for 2 hours. Fold increase in ^{14}C-actetate flux into lipid-soluble fraction was determined (flox, n = 3; $ACC^{\Delta LysM}$, n = 4)
Significance determined by two-tailed Student's t-test (E,F), one-tailed Mann-Whitney test (G,H) or One-Way ANOVA (C,D). * $P < 0.05$, ** $P < 0.01$, *** $P < 0.001$, **** $P < 0.0001$

Supplementary Table 1. Primer Sequences for RT-qPCR

Gene	Forward Primer	Reverse Primer	Source
Mus musculus			
Acaca	GTCTGCTGGGAAGTTAATCCAG	TCCTGCAGCTCTAGCAGAGG	(*25*)
Acacb	ACAGAGATTTCACCGTCGCGT	CGCAGCGATGCCATTGT	(*25*)
Il6	ACAACGATGATGCACTTGCAG	GCATTGGAAATTCGGGTAGGAA	(*62*)
Il1b	CAAAATACCTGTGGCCTTGG	TACCAGTTGGGGAACTCTGC	(*7*)
Il18	GACTCTTGCGTCAACTTCAAGG	CAGGCTGTCTTTTGTCAACGA	Primer Bank
Arg1	AAGACAGCAGAGGAGGTGAAGAG	TGGGAGGAGAAGGCGTTTGC	(*7*)
Chil3	CTTCCACAGGAGCAGGAATC	GCTCCATGGTCCTTCCAGTA	(*6*)
Ifng			
Nos2	CCTGGTACGGGCATTGCT	GCTCATGCCTCCTT	(*7*)
B2m	ATTCACCCCCACTGAGACTG	TGCTATTTCTTTCTGCGTGC	(*6*)
Hprt	TGAAGTACTCATTATAGTCAAGGGCA	CTGGTGAAAAGGACCTCTCG	(*29*)
Mrc1			Primer Bank
Retnla	CCAATCCAGCTAACTATCCCTCC	CCAGTCAACGAGTAAGCACAG	Primer Bank
Il12a	CTGTGCCTTGGTAGCATCTATG	GCAGAGTCTCGCCATTATGATTC	Primer Bank
Il12b	TGGTTTGCCATCGTTTTGCTG	ACAGGTGAGGTTCACTGTTTCT	Primer Bank
Aldoc	TCTCTCTTGGGATCAGGGGG	CAGGTGAACCCTTCCTCCAC	(*6*)
Gapdh	TGAAGGGTGGAGCCAAAAGG	ACTTGGCAGGTTTCTCCAGG	(*6*)
Pfkfb3	AACGGATGTCTCCCGGTTTC	TGGTATGGAGGCTGCTCTCT	Primer Bank
Acly	TGATGGGAGAAGTTGGGAAG	ATCAGCTCGGGACTCAGAAA	Primer Bank
Fasn	GGAGGTGGTGATAGCCGGTAT	TGGGTAATCCATAGAGCCCAG	Primer Bank
Acat2	CCCGTGGTCATCGTCTCAG	GGACAGGGCACCATTGAAGG	Primer Bank
Mvd	ATGGCCTCAGAAAAGCCTCAG	TGGTCGTTTTTAGCTGGTCCT	Primer Bank
Hmgcs1	AACTGGTGCAGAAATCTCTAGC	GGTTGAATAGCTCAGAACTAGCC	Primer Bank
Lss	TCGTGGGGGACCCTATAAAAC	CGTCCTCCGCTTGATAATAAGTC	Primer Bank
Dhcr24	CTCTGGGTGCGAGTGAAGG	TTCCCGGACCTGTTTCTGGAT	Primer Bank
Cd80	GCTGTGTCGTTCAAAAGAAGGA	TGGGAAATTGTCGTATTGATGCC	Primer Bank
Cd86	GAGCTGGTAGTATTTTGGCAGG	GGCCCAGGTACTTGGCATT	Primer Bank
Il10	ACTACCAAAGCCACAAGGCA	TGGCAACCCAAGTAACCCTTA	(*6*)
Homo sapiens			
ACACA	CATGCGGTCTATCCGTAGGTG	GTGTGACCATGACAACGAATCT	Primer Bank
ACACB	AGAAGACAAGAAGCAGGCAAAC	GTAGACTCACGAGATGAGCCA	Primer Bank
IL6	ACTCACCTCTTCAGAACGAATTG	CCATCTTTGGAAGGTTCAGGTTG	Primer Bank

IL1B	AGCTACGAATCTCCGACCAC	CGTTATCCCATGTGTCGAAGAA	Primer Bank
RPS18	GCGGCGGAAAATAGCCTTTG	GATCACACGTTCCACCTCATC	Primer Bank

Data S1. Lipidomics_Data

Summary data for lipidomics experiment from unstimulated or LPS-treated flox and ACC$^{\Delta LysM}$ BMDMs.

Data S3. Source_Data_File

Source Data for figures.

Data S2. Uncropped_Gels_Blots: Summary PDF of uncropped gels and blots.

Macrophage acetyl-CoA carboxylase regulates acute inflammation through control of glucose and lipid metabolism

Scott Yeudall[1], Clint M Upchurch[1], Philip V Seegren[1], Caitlin M Pavelec[1,2], Jan Greulich[3], Michael C Lemke[1], Thurl E Harris[1], Bimal N Desai[1], Kyle L Hoehn[1,4], and Norbert Leitinger[1,2]

[1] Department of Pharmacology,
[2] Robert M Berne Cardiovascular Research Center,
University of Virginia School of Medicine, Charlottesville, Virginia 22908

[3]Environmentally-Induced Cardiovascular Degeneration, Clinical Chemistry and Laboratory Diagnostics, Medical Faculty, University Hospital and Heinrich-Heine University Düsseldorf, 40225 Düsseldorf, Germany

[4]School of Biotechnology and Biomolecular Sciences,
University of New South Wales, Sydney, Australia 2052.

Uncropped blots and gels

Full unedited blots for Figure 1C

MW

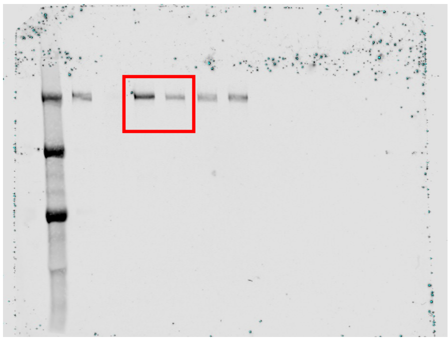

pan-ACC (CST #3676, C83B10)

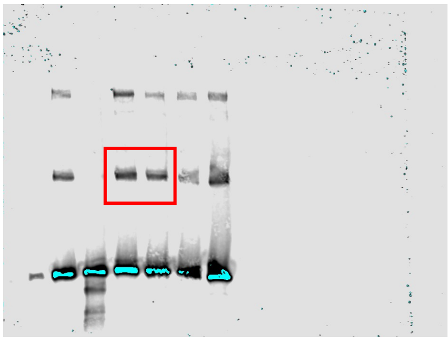

IRDye-800CW conjugated streptavidin (Licor # 926-32230)

IR800-conjugated avidin (for biotinylated proteins) for pyruvate carboxylase

IR800-conjugated avidin (for biotinylated proteins) for propionyl-coA carboxylase (blue indicates saturation of detection on Licor instrument)

Samples after streptavidin pulldown of cell lysates – lanes alternate flox and $ACC^{\Delta LysM}$ BMDMs. Lanes 3 and 4 are shown in figure 1C.

Full unedited blots for Figure 4C

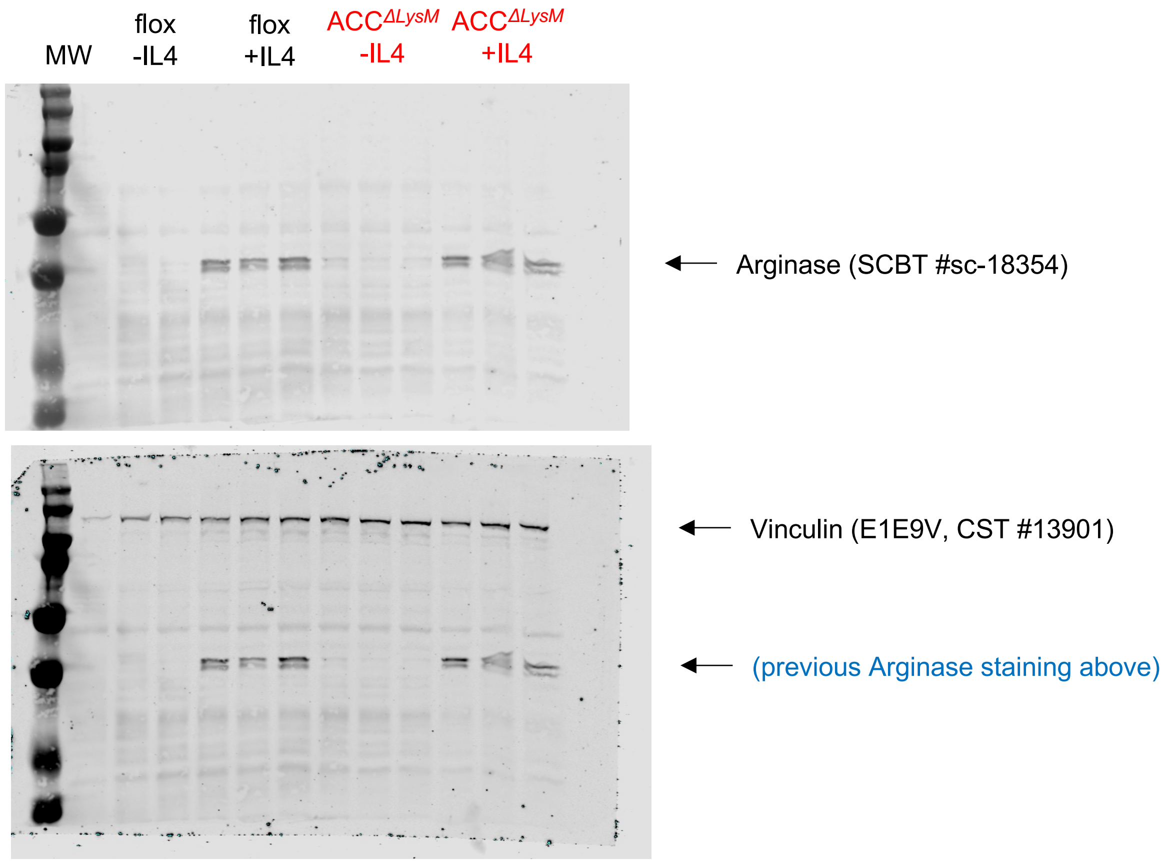

Full unedited blots for Supplementary Figure 1E

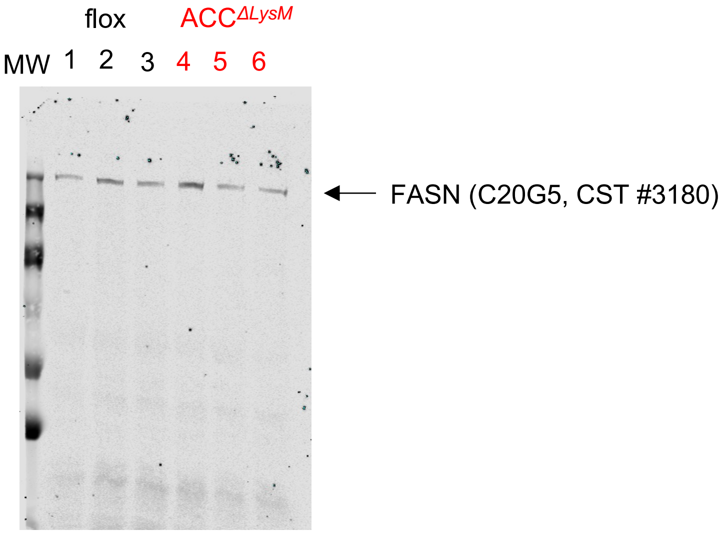

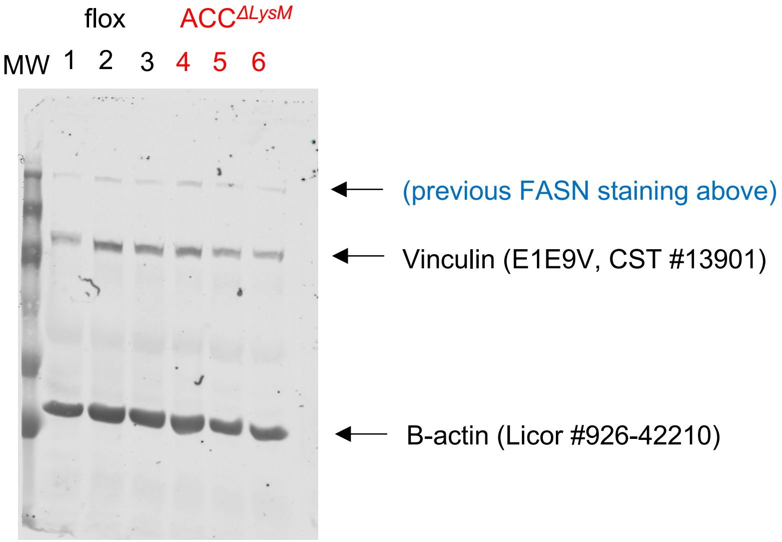

Lanes 3 and 4 displayed in Supplementary Figure 1E

Full unedited blots for Supplementary Figure 1K

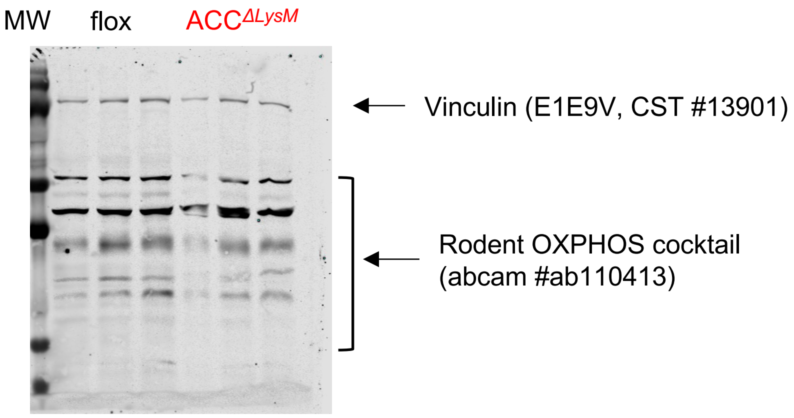

Full unedited blots for Supplementary Figure 2B

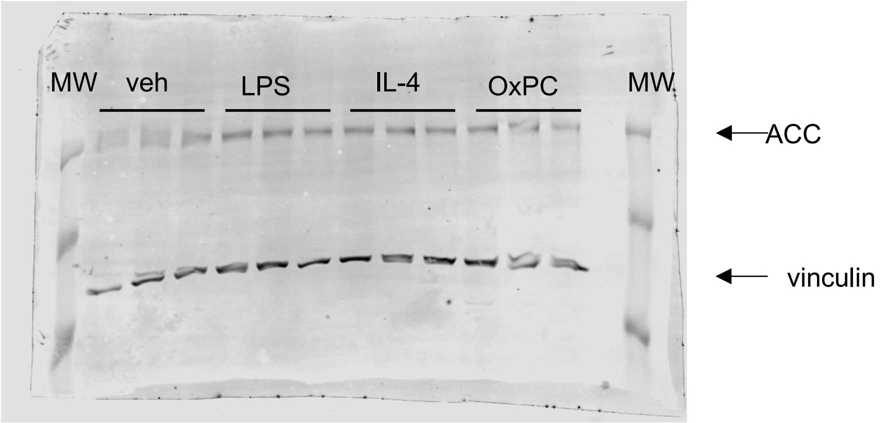

Full unedited blots for Supplementary Figure 2E

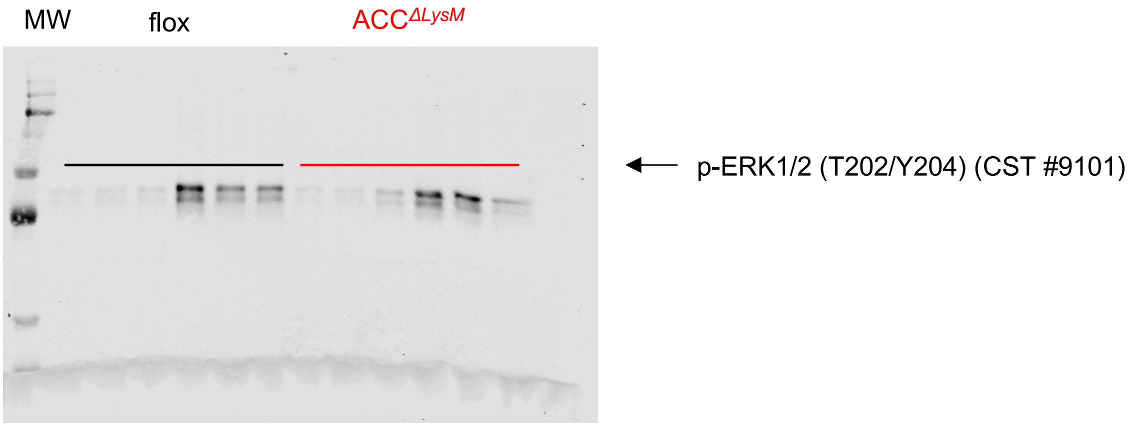

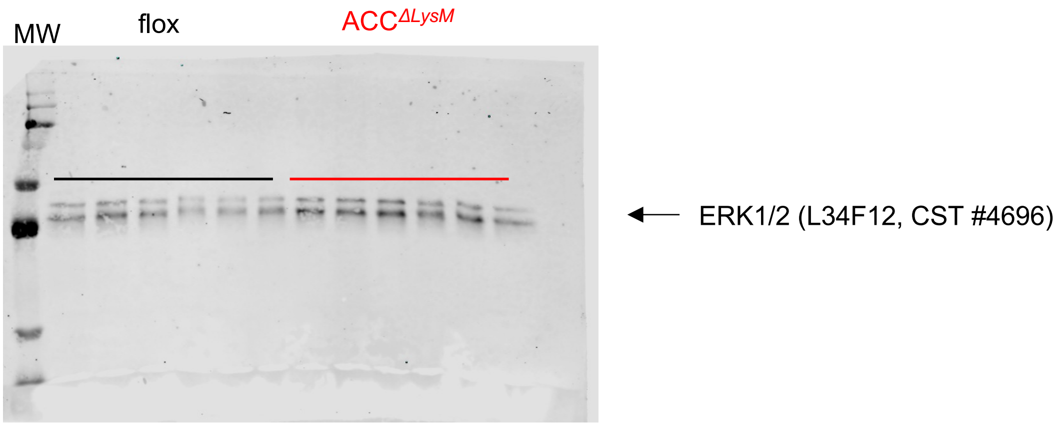

Full unedited blots for Supplementary Figure 2F

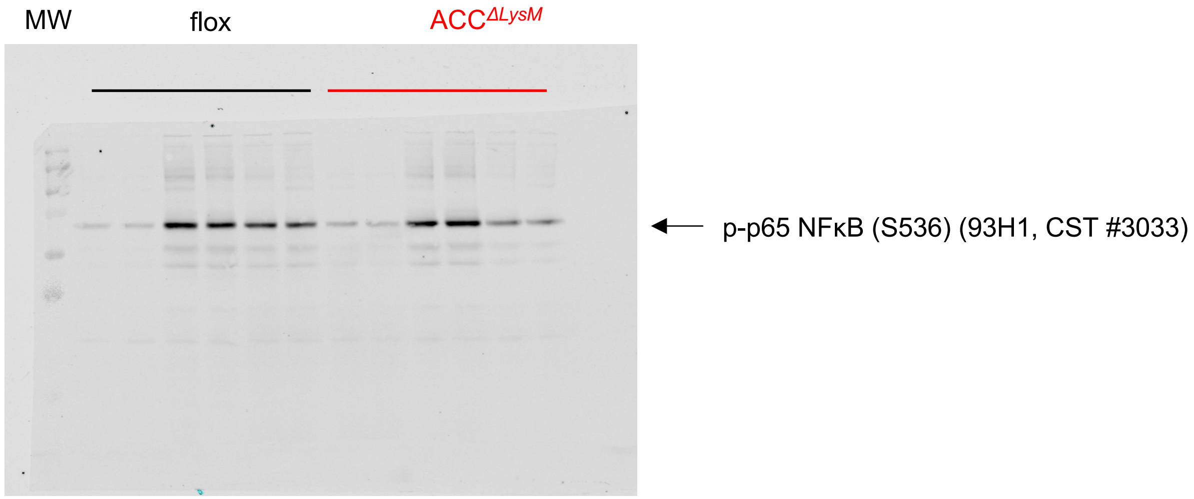

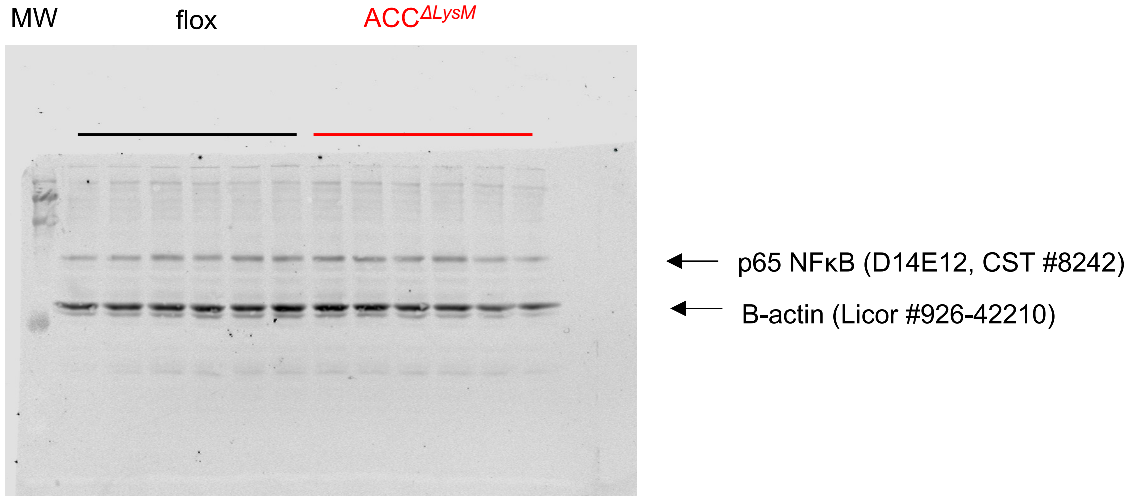

Full unedited blots for Supplementary Figure 2G

cytoplasmic

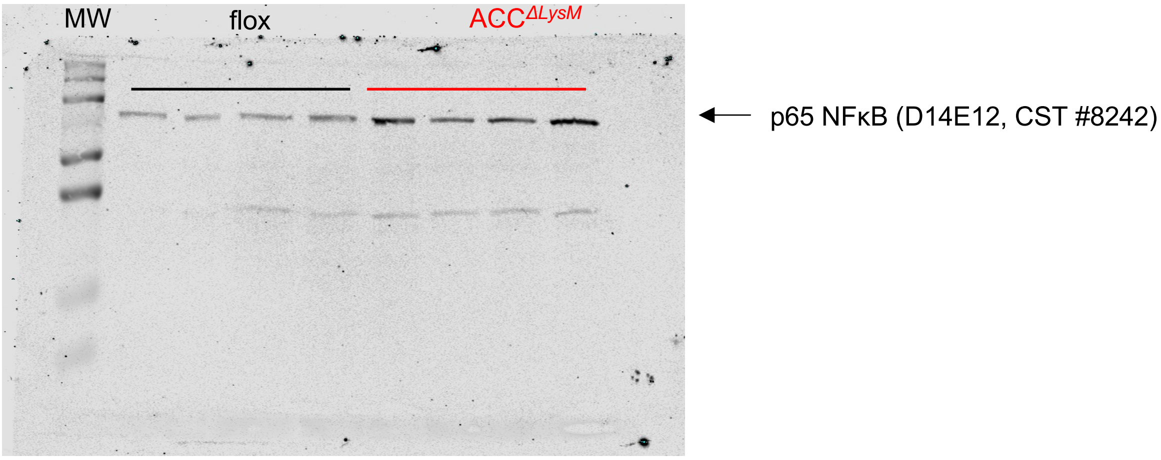

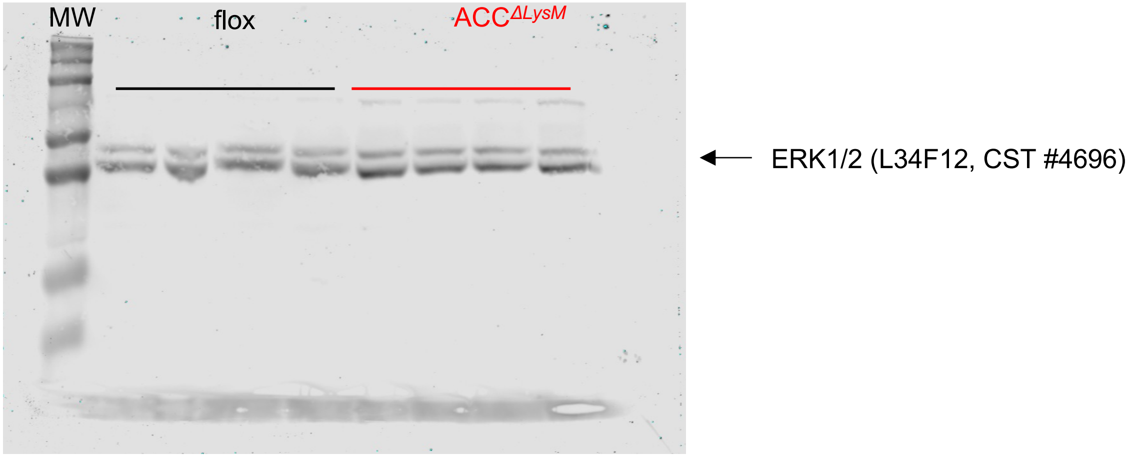

Full unedited blots for Supplementary Figure 2G

nuclear

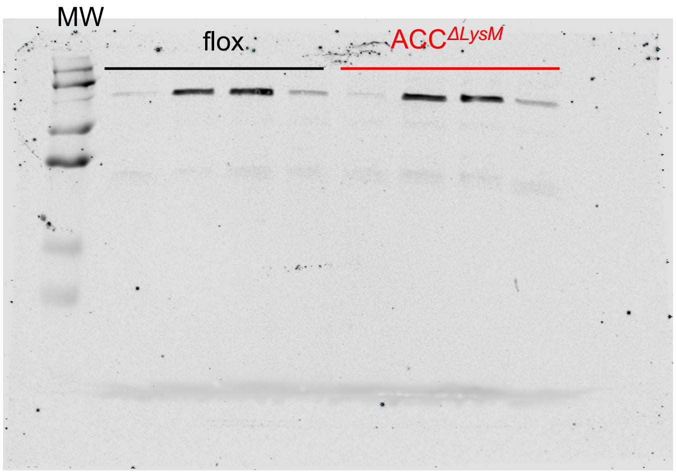

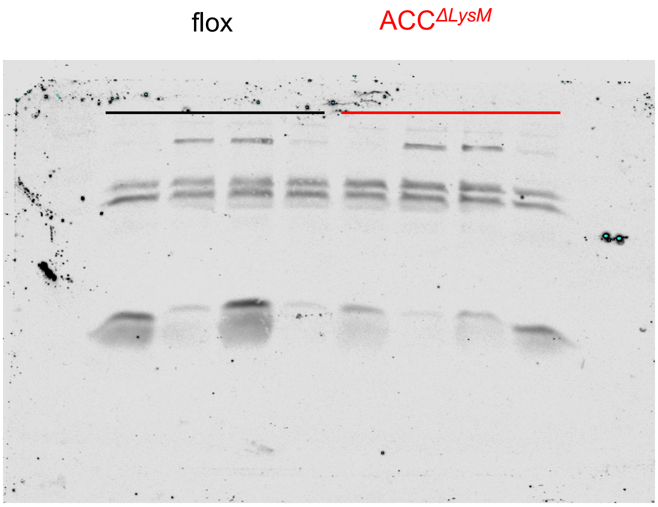

Full unedited blots for Supplementary Figure 3B

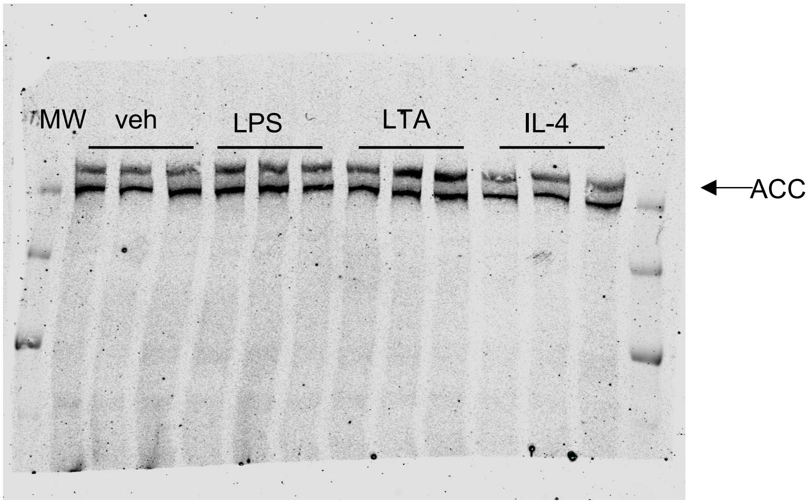

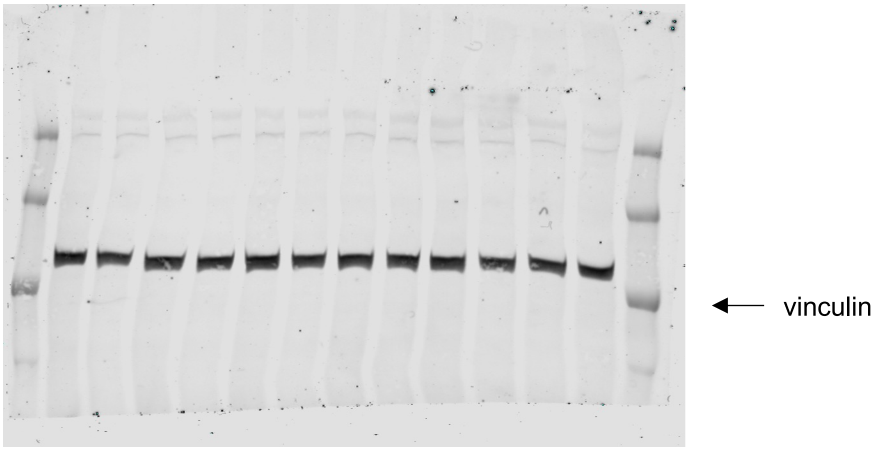

Full unedited blots for Supplementary Figure 3D

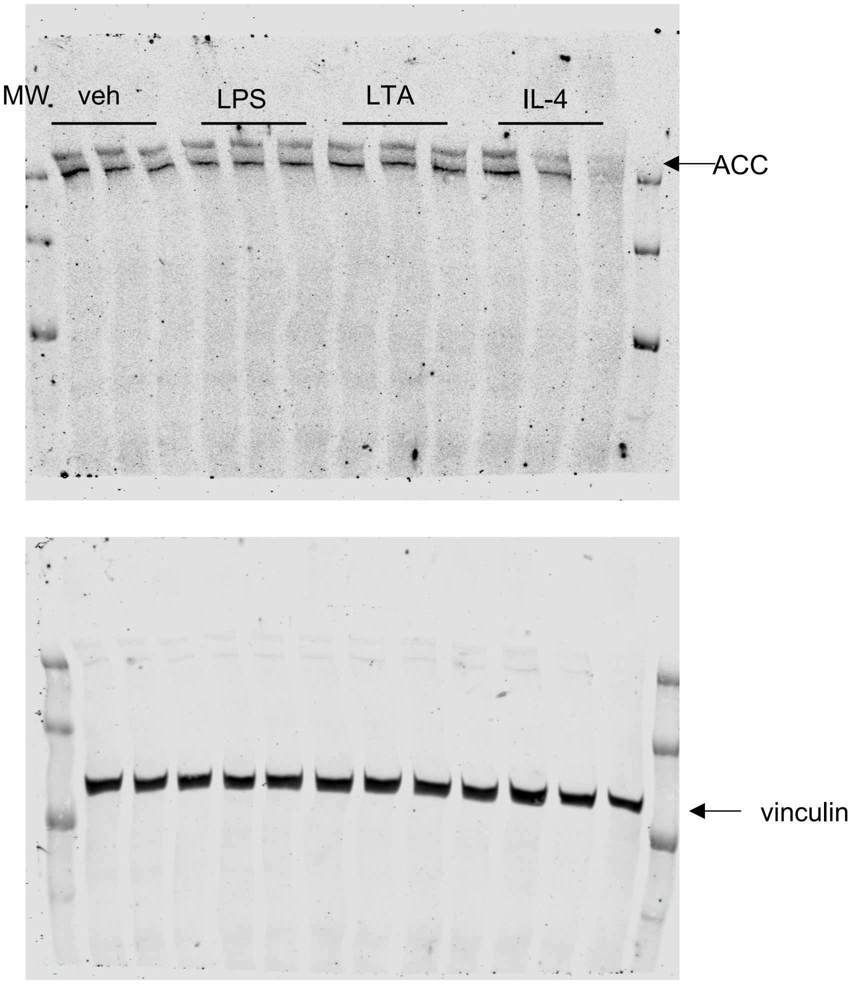

Full unedited blots for Figure S4B

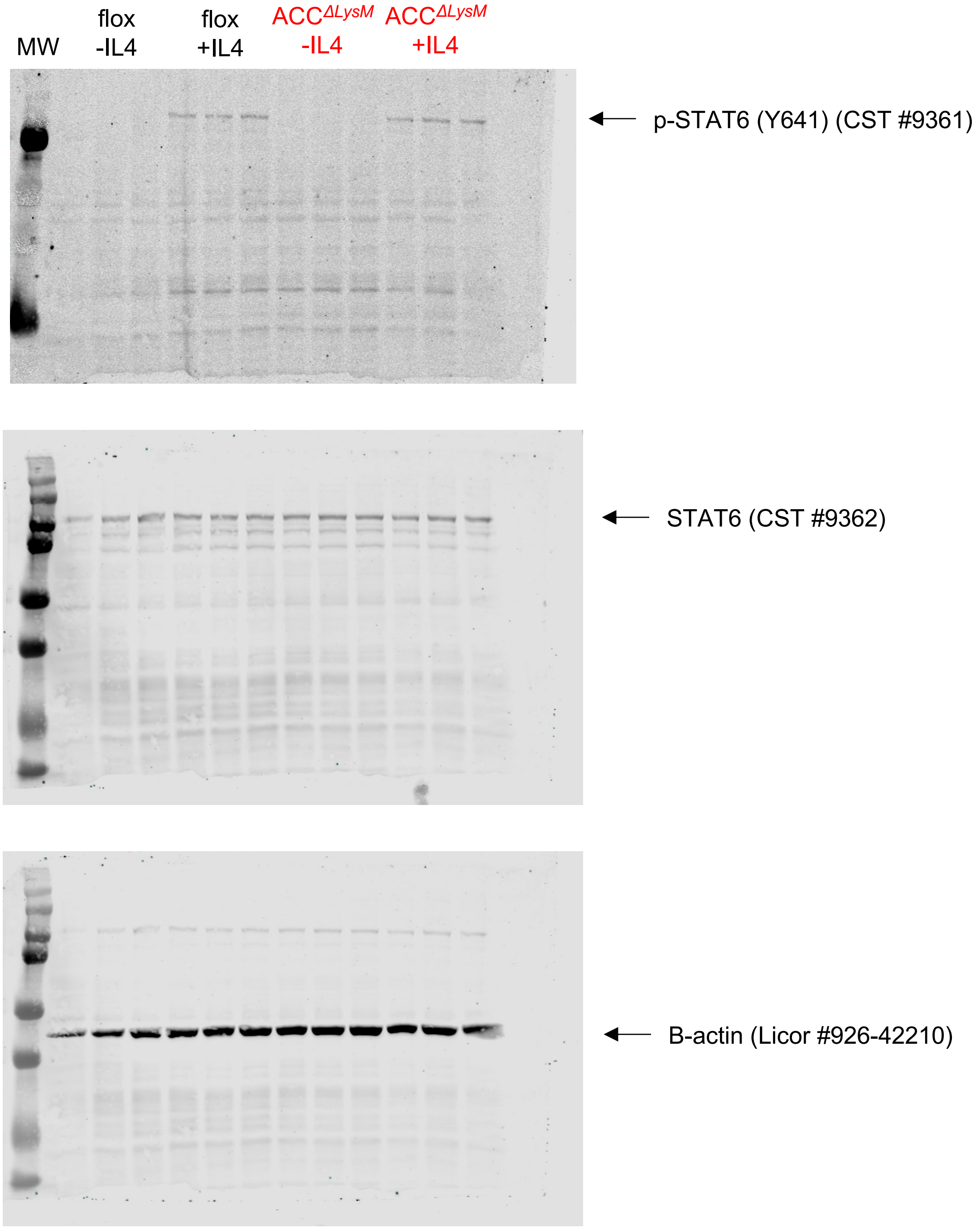

Full unedited blots for Figure S5C

Cell-surface biotin pulldown

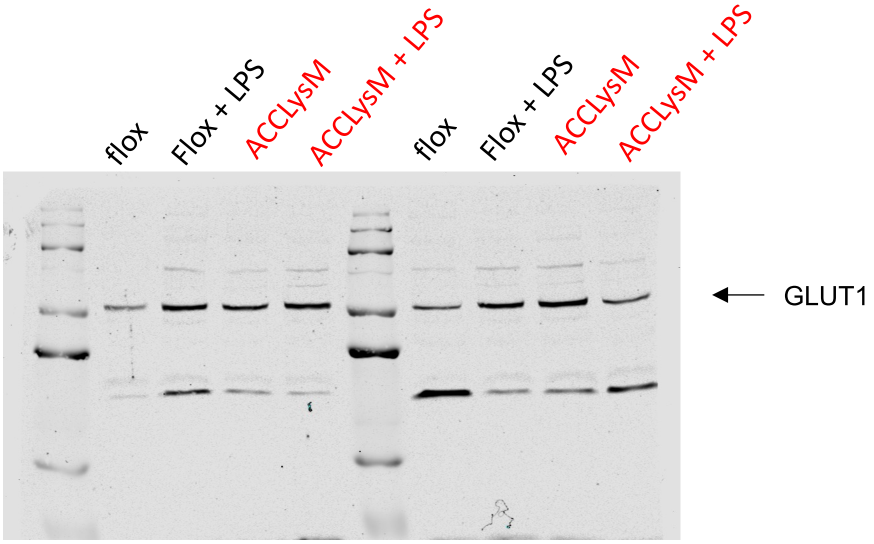

Input: whole cell lysate

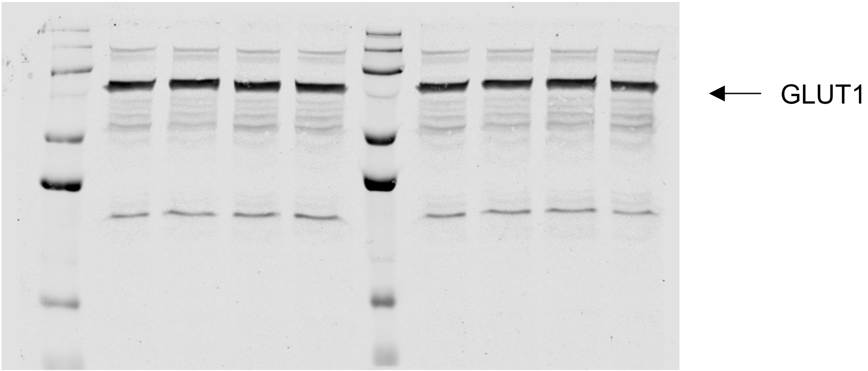

Full unedited gels for Supplementary Figure 1A – DNA gels

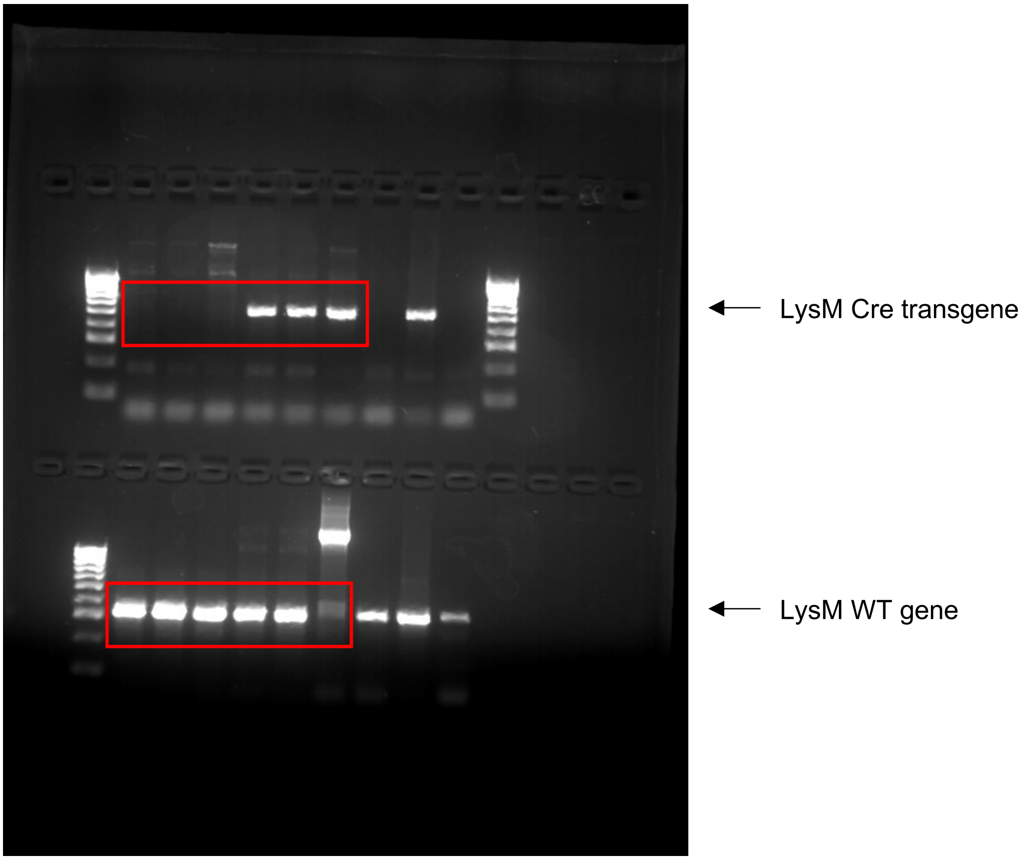

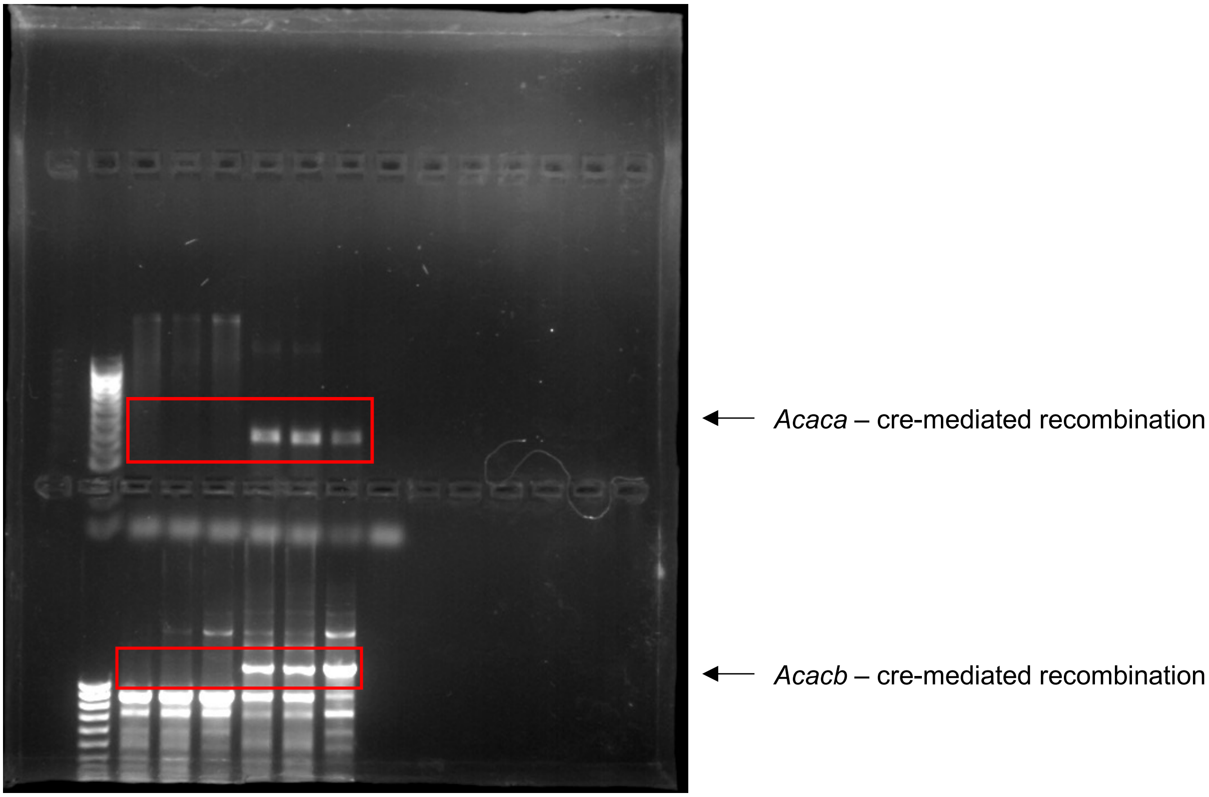

Supplement: Supplementary file 1 — Figs. S1 to S6 Table S1 Data S2. Uncropped_Gels_Blots: Summary PDF of uncropped gels and blots. [file sciadv.abq1984_sm.pdf]
